# Effectiveness of Internet-Based Multicomponent Interventions for Patients and Health Care Professionals to Improve Clinical Outcomes in Type 2 Diabetes Evaluated Through the INDICA Study: Multiarm Cluster Randomized Controlled Trial

**DOI:** 10.2196/18922

**Published:** 2020-11-02

**Authors:** Yolanda Ramallo-Fariña, Miguel Angel García-Bello, Lidia García-Pérez, Mauro Boronat, Ana M Wägner, Leticia Rodríguez-Rodríguez, Pedro de Pablos-Velasco, Ignacio Llorente Gómez de Segura, Himar González- Pacheco, Montserrat Carmona Rodríguez, Pedro Serrano-Aguilar, INDICA Team

**Affiliations:** 1 Canary Islands Health Research Institute Foundation (FIISC) Tenerife Spain; 2 Research Network on Health Services in Chronic Diseases (REDISSEC) Madrid Spain; 3 Evaluation Unit (SESCS) Canary Islands Health Service (SCS) Tenerife Spain; 4 Center for Biomedical Research of the Canary Islands (CIBICAN) Tenerife Spain; 5 Department of Endocrinology and Nutrition Insular University Hospital Las Palmas de Gran Canaria Spain; 6 University Institute for Biomedical and Health Research (IUIBS) University of Las Palmas de Gran Canaria (ULPG) Las Palmas de Gran Canaria Spain; 7 Department of Endocrinology and Nutrition Doctor Negrín University Hospital Las Palmas de Gran Canaria Spain; 8 Department of Endocrinology and Nutrition Nuestra Señora de la Candelaria University Hospital Santa Cruz de Tenerife Spain; 9 Health Technology Assessment Agency Instituto de Salud Carlos III Madrid Spain; 10 See Author´s Contributions Section Santa Cruz de Tenerife Spain

**Keywords:** behavior modification, primary care, type 2 diabetes mellitus, patients adherence, eHealth

## Abstract

**Background:**

Type 2 diabetes mellitus (T2DM) is a chronic disease in which health outcomes are related to decision making by patients and health care professionals.

**Objective:**

This study aims to assess the effectiveness of internet-based multicomponent interventions to support decision making of all actors involved in the care of patients with T2DM in primary care.

**Methods:**

The INDICA study is an open, community-based, multicenter trial with random allocation to usual care or the intervention for patients, the intervention for health care professionals in primary care, or the combined intervention for both. In the intervention for patients, participants received an educational group program and were monitored and supported by logs, a web-based platform, and automated SMS. Those in the intervention for professionals also received an educational program, a decision support tool embedded in the electronic clinical record, and periodic feedback about patients’ results. A total of 2334 people with T2DM, regardless of glycated hemoglobin (HbA_1c_) levels and without diabetes-related complications, were included. The primary end point was change in HbA_1c_ level. The main analysis was performed using multilevel mixed models.

**Results:**

For the overall sample, the intervention for patients attained a significant mean reduction in HbA_1c_ levels of ‒0.27 (95% CI ‒0.45 to ‒0.10) at month 3 and ‒0.26 (95% CI ‒0.44 to ‒0.08) at month 6 compared with usual care, which remained marginally significant at month 12. A clinically relevant reduction in HbA_1c_ level was observed in 35.6% (191/537) of patients in the intervention for patients and 26.0% (152/586) of those in usual care at month 12 (*P*=.006). In the combined intervention, HbA_1c_ reduction was significant until month 18 (181/557, 32.6% vs 140/586, 23.9%; *P*=.009). Considering the subgroup of patients uncontrolled at baseline, all interventions produced significant reductions in HbA_1c_ levels across the entire study period: ‒0.49 (95% CI ‒0.70 to ‒0.27) for the intervention for patients, ‒0.35 (95% CI ‒0.59 to ‒0.14) for the intervention for professionals, and ‒0.35 (95% CI ‒0.57 to ‒0.13) for the combined intervention. Differences in HbA_1c_ for the area under the curve considering the entire period were significant for the intervention for patients and the combined intervention compared with usual care (*P*=.03 for both). Compared with usual care, the intervention for professionals and the combined intervention had significant longer-term reductions in systolic and diastolic blood pressure.

**Conclusions:**

In uncontrolled patients, the intervention for patients at baseline provided clinically relevant and significant longer-term reductions of HbA_1c_ levels. The intervention for professionals and combined intervention also improved the cardiovascular risk profile of patients.

**Trial Registration:**

ClinicalTrials.gov NCT01657227; https://clinicaltrials.gov/ct2/show/NCT01657227

## Introduction

### Background

Type 2 diabetes mellitus (T2DM) is a chronic condition in which long-term health outcomes are related to patients’ adherence to lifestyle modifications and pharmacologic treatments. Other stakeholders, such as relatives and primary health care professionals, are also involved in guiding patients’ decisions.

Although the prevalence of T2DM in the Canary Islands is slightly higher than the average in Spain [1], the incidence of chronic diabetes-related complications [2,3] and mortality [4] is much greater. This occurs despite a continuous increase in diabetes-related public expenditure [5].

Regardless of the widespread availability of evidence-based clinical practice guidelines (CPGs) to care for T2DM, patients’ access to effective educational interventions [6] and adherence to self-management activities remains limited internationally [7].

To address these unmet needs, many publications have reported on the effectiveness of using information and communications technology (ICT) applications to support decision making by patients and professionals [8-12], reporting favorable short-term effects on blood glucose control [11,12]. The effectiveness of other biological, cognitive, behavioral, or emotional outcome measures remains controversial [11]. Few large randomized controlled trials (RCTs) have assessed the long-term effectiveness of multicomponent ICT-based interventions, not only for patients but also for all stakeholders involved in diabetes management.

### Objectives

The INDICA study is a cluster RCT conducted in the Canary Islands that assesses the effectiveness and cost-effectiveness of multicomponent interventions to support decision making for the main actors involved in the management of T2DM (patients, relatives, and primary health care professionals) in a large number of primary health care practices (PHCPs) [13]. We hypothesized that combining conventional educational activities with different ICT-based decision support tools would efficiently improve health outcomes in patients with T2DM. The main purpose of this study is to evaluate the long-term clinical effectiveness (24 months) of these multicomponent interventions compared with usual care on glycated hemoglobin (HbA_1c_).

## Methods

### Study Design

The INDICA study is an open, community-based pragmatic, multicenter, clinical controlled trial with random allocation by clusters to usual care or to one of the following 3 interventions of knowledge transfer and behavior modification:

- Group 1 included interventions for patients and a family member (intervention for patients)
- Group 2 included interventions for health care professionals (physicians and nurses) at primary care (intervention for professionals)
- Group 3 combined the interventions for patients and professionals (combined intervention)

In the usual care or control group, neither patients or families nor physicians or nurses received any additional educational or supporting activities beyond the usual activities provided by the PHCP. The full study protocol has been reported elsewhere [13].

### Study Participants

The INDICA study included patients with T2DM aged between 18 and 65 years, diagnosed at least 1 year before study enrollment, without diabetes-related complications, and who regularly used a mobile phone (Textbox 1 provides more details).

**Patients’ inclusion and exclusion criteria.**

Patient inclusion criteria:

- Patients with type 2 diabetes mellitus diagnosed at least 1 year before study enrollment
- Aged between 18 and 65 years
- Formal consent to participate in the study
- Regular usage of mobile phone

Patient exclusion criteria:

- Chronic kidney disease ≥ stage 3b, as defined by the National Kidney Foundation’s Kidney Disease Outcomes and Quality Improvement Initiative, urinary albumin to creatinine ratio ≥ 300 mg/g, or urinary protein excretion ≥ 300 mg/24 hours
- Acute coronary syndrome (documented angina or myocardial infarction) or stroke in the last 6 months or class III or IV heart failure, according to the New York Heart Association
- Proliferative diabetic retinopathy or clinically significant diabetic macular edema requiring previous treatment with retinal photocoagulation, vitrectomy, or intravitreal injections of antivascular endothelial growth factor or triamcinolone acetonide 6 months before study inclusion
- Uncorrected severe hearing or visual impairment or corrected visual acuity ≤ 20/40 by any cause
- Diabetic foot with ulcers ≥ 2 according to the Wagner scale
- Liver cirrhosis
- Cancer, unless disease free 5 years after diagnosis
- Other terminal illnesses
- Intellectual retardation, dementia, and psychotic diseases
- Active substance abuse, alcohol, or drugs (must be sober for 1 year)
- Pregnancy
- Insufficient (Spanish) language skills
- Physical disability limiting participation in group education activities
- Concurrent participation in another clinical trial or any other investigational study

The family care unit (FCU) in each PHCP, comprising a family physician and a nurse responsible for the same set of patients, was the unit of recruitment. FCUs either planning or awaiting placement changes among PHCP in the first 6 months after project initiation were excluded.

All PHCPs included had to have at least eight FCUs and the availability of appropriate places to provide educational group sessions.

### Setting and Recruitment

PHCPs were recruited in 4 Canary Islands (Tenerife, Gran Canaria, Lanzarote, and La Palma). FCUs were randomly selected from all consenting FCUs at each PHCP. The electronic clinical records (ECRs) of all potentially eligible patients in all selected FCUs were screened to verify inclusion and exclusion criteria. Finally, eligible patients were randomly selected per FCU.

### Random Assignment

Randomization was performed at different levels. First, 3 different strata were created according to the geographical areas in the more populated islands (Tenerife and Gran Canaria). Second, 4 PHCP (clusters) were randomly allocated to every geographical stratum, and block permutation was used to assign PHCPs to the study arms (in total 12 PHCPs for each island), with PHCP as the sampling unit. La Palma and Lanzarote (less populated islands) were geographically divided into 4 zones with only 1 eligible PHCP available in each zone, which was randomly assigned to one of the study arms. On every island, all arms were equally distributed. A total of 6 FCUs were randomly selected from all those consenting to participate in each PHCP. Furthermore, 15 patients were randomly selected from all patients fulfilling the inclusion criteria and consenting to participate in each FCU. Exceptionally, more than 6 FCUs or more than 15 patients per FCU were selected to recruit 90 patients at every PHCP.

FCU and patient randomizations were performed by simple generation from a list of random numbers.

Cluster allocation avoids contamination bias among participants, also facilitating logistics in group interventions.

### Interventions

#### Patient Interventions

Patients recruited to the intervention for patients and combined intervention groups received a complex intervention of knowledge transfer and behavior modification, informed by conceptual frameworks of behavioral change [14]. Key determinants of behavior change suggested by Michie et al [14] were considered for intervention design and implementation, including social and professional role and identity, knowledge, skills, beliefs about capabilities, beliefs about consequences, motivation and goals, memory, attention and decision processes, environmental context and resources, social influences, emotion, and action planning. Linked to these construct domains, interventions included all techniques judged as effective by the same authors [14], combining (1) a conventional group educational program with a set of 8 quarterly 3-hour group sessions; (2) monitoring of physical activity, diet, drug adherence, mood, blood pressure, and blood glucose readings by daily usage of paper workbooks, complemented by weekly access to a website to download paper workbook data (Multimedia Appendix 1); and (3) continuous personalized feedback by semiautomated mobile phone messages based on the results from the website.

#### Interventions for Primary Care Professionals

Primary care professionals recruited to the intervention for professionals and combined intervention groups received a complex intervention of knowledge transfer and decision support, partially addressing the determinants of behavior change suggested by Michie et al [14] for its design and implementation, including only techniques to improve skills, environmental changes, prompts and cues by means of electronic clinical guidelines linked to the ECR, processes for encouraging and supporting doctors and nurses, persuasive communication, and periodic feedback on outcomes compared with other colleagues. The interventions combined (1) an educational and interactive group program of 2 sessions to update clinical management and promote patient-centered care; (2) an automated decision aid tool based on a CPG for T2DM, embedded into the ECR (Multimedia Appendix 2); and (3) monthly computerized graphic feedback, displaying a set of processes and outcome indicators for all patients with T2DM of the corresponding FCU.

To maintain the fidelity of interventions, a manual was developed for each intervention. Furthermore, all group sessions were recorded and reviewed.

**Figure 1:**
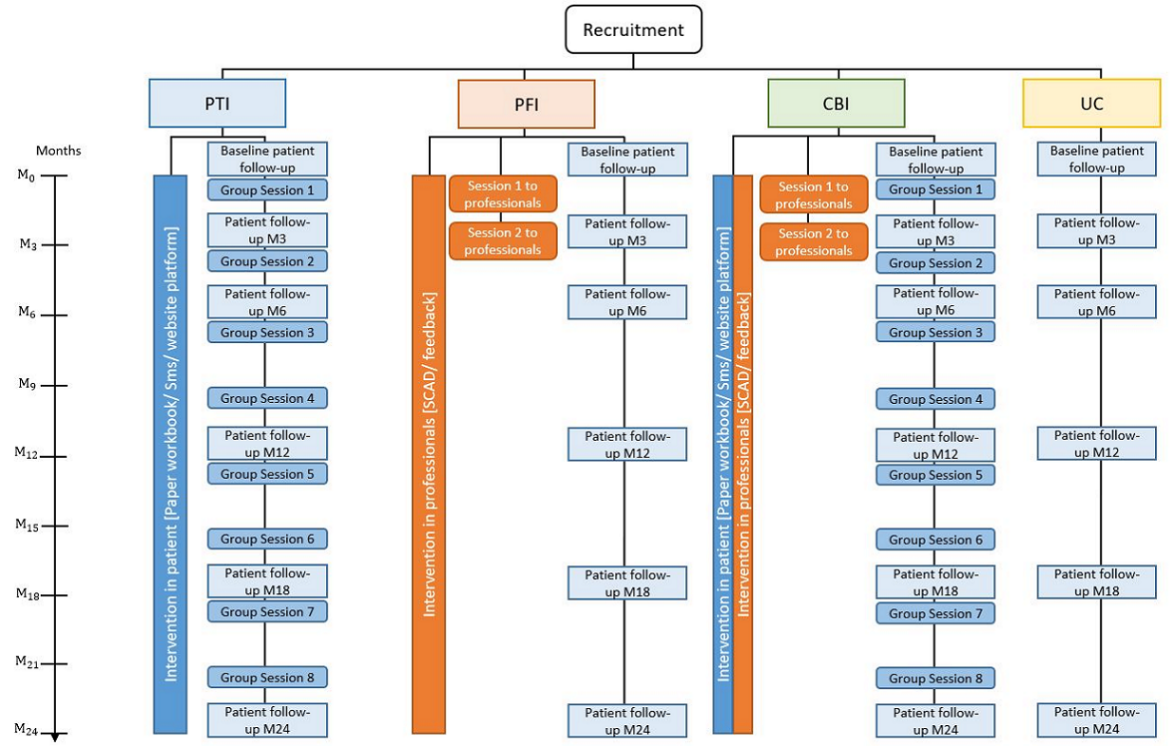
Arm’s intervention timeline and follow-up points.

Both interventions were applied during the 2 years of follow-up (Figure 1).

### Duration of Fieldwork

Fieldwork took place between February 2013 and October 2016. The first year was devoted to the recruitment of patients and health care providers and the following 2 years to the intervention and follow-up. As interventions were maintained over time, the intervention and follow-up periods overlapped.

### Outcomes

#### Primary End Point

The primary outcome was the mean change in HbA_1c_ levels from baseline to 24 months of follow-up. HbA_1c_ was also measured at 3, 6, 12, and 18 months. We considered a change in HbA_1c_ of 0.4% as clinically significant [15], just between the thresholds of 0.3% reported by National Institute for Health and Care Excellence [16] and 0.5% by the United Kingdom Prospective Diabetes Study [17].

#### Secondary End Points

BMI, weight, waist circumference, waist-to-hip ratio, systolic blood pressure (SBP), and diastolic blood pressure (DBP) were also assessed at baseline and after 3, 6, 12, 18, and 24 months. Total, high-density lipoprotein (HDL), and low-density lipoprotein (LDL) cholesterol, triglycerides, and fasting serum glucose were assessed at baseline and after 6, 12, and 24 months. Serum creatinine and glomerular filtration rate were measured at baseline and at 12 and 24 months. Demographic data and disease history were recorded at baseline. Health status and current medications were also recorded at each follow-up.

### Statistical Analysis

The main analysis for primary and secondary end points were multilevel mixed models including the baseline value of the dependent variable and the time elapsed since diagnosis (in years) as covariates. The null hypothesis for each end point is that the mean change with regard to the usual care arm and the interactions between each arm and time (follow-up) are the same across arms and equal to zero. The alternative hypothesis is that the changes are not equal to zero. First-level variables are those corresponding to each measurement along follow-up (repeated time measurements), the second level includes patients’ variables, and third-level variables correspond to PHCPs. The mean change was estimated at the observation level. The effect that identifies the intervention arm was considered fixed for the PHCPs, whereas the intercept was considered random. The model also included an interaction term between arm and month, allowing for differences in the intervention effect between follow-up assessments [18]. In addition, to summarize the global treatment effect throughout the whole study period, differences were also calculated for the area under the curve (AUC) of HbA_1c_ and other continuous variables between the different interventions and the usual care group. Furthermore, we examined whether the most intensive intervention, the combined intervention group (intervention for patients plus intervention for professionals), was better than the intervention for patients and intervention for professionals groups on their own.

The adjusted estimated mean was calculated for each moment of follow-up compared with baseline, and its significance was calculated using the model already set out.

A post hoc analysis was performed for the primary end point, HbA_1c_, considering the patient subsample with baseline HbA_1c_ higher than 7%.

To accommodate missing values in the effect analyses, the multiple imputation procedure in Stata 15.0 software (Stata Corporation) was used [19], with results based on 100 imputed data sets. This procedure saves cases for the analysis and can be considered an intention-to-treat analysis. Analysis under multiple imputation is valid for randomly missed data [20]. The model of imputation used and further details on data analysis are outlined in Multimedia Appendix 3. A threshold of .05 was used to define the statistical significance of those tests.

### Sample Size Calculation

We estimated the sample size requirement of 448 patients per study arm to detect an absolute difference in HbA_1c_ of 0.4%, assuming a common standard deviation of 1.4% [15], a two-tailed power of 90%, an alpha of .05, and an adjustment for clustering of patients within the FCU by the design effect [21], 15 patients per FCU, and an intraclass correlation coefficient (ICC) of 0.01 (interquartile range 0-0.032) [22]. The intraclass correlation within PHCPs was insignificant as they are formed of several FCUs sharing administrative management and some additional services whose potential effects were already controlled by means of the stratification. Despite this consideration, the sample size was increased by an additional 30% to accommodate for expected losses to follow-up and to warrant the presence of each study arm in all islands. Hence, we aimed to obtain a total sample size of 2330.

### Ethics Approval and Consent to Participate

All participants provided written informed consent. The scientific and ethics committees of both the University Hospital of Canarias and the University Hospital Nuestra Señora de la Candelaria approved the study protocol. The study was performed in accordance with Good Clinical Practice standards, applicable local regulatory requirements, and the recommendations of the Declaration of Helsinki.

## Results

### Study Participants

**Figure 2:**
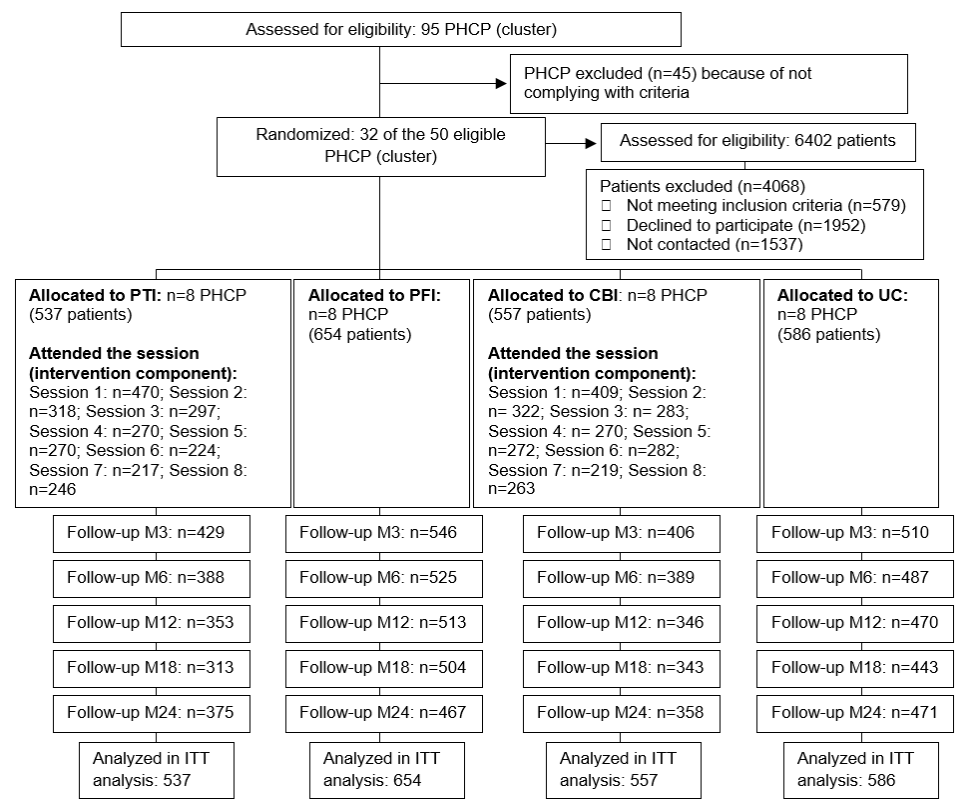
CONSORT (Consolidated Standards of Reporting Trials) flow diagram.

A total of 32 PHCPs with a mean of 6.6 (SD 0.9) FCUs were included (211 professionals), with 8 PHCPs allocated to each of the 4 study arms. Every PHCP enrolled a mean of 72.9 (SD 14.1) patients (12 patients per FCU), totaling 2334 patients. Figure 2 shows the flowchart for the patients taking part.

Table 1 shows the patients’ baseline characteristics according to the intervention assignment. The mean age of the whole population was 55.7 (SD 7.1) years, with 51.9% (1212/2334) being women. The mean basal HbA_1c_ value was 7.3% (SD 1.5). Overall, 53.4% (1246/2334) of patients had HbA_1c_ levels within the accepted therapeutic goal (≤7%). There were no statistically significant differences among the groups in terms of their baseline characteristics.

**Table 1:** Baseline characteristics of patients.

Characteristics		PTI^a^ (n=537)	PFI^b^ (n=654)	CBI^c^ (n=557)	UC^d^ (n=586)
Age (years), mean (SD)		55.9 (7.0)	56.2 (7.0)	55.5 (7.1)	55.2 (7.3)
Gender (male), n (%)		284 (52.9)	288 (44.0)	264 (47.4)	286 (48.8)
**Smoking status, n (%)**					
	Current smokers	114 (21.2)	156 (23.9)	109 (19.6)	145 (24.7)
	Former smokers	223 (41.5)	280 (42.8)	225 (40.4)	240 (41.0)
	Nonsmoker	200 (37.2)	218 (33.3)	223 (40.0)	201 (34.3)
**Education, n (%)**					
	Primary or less	323 (60.2)	409 (62.5)	347 (62.3)	379 (64.7)
	High school	159 (29.6)	176 (26.9)	147 (26.4)	157 (26.8)
	Bachelor’s degree or higher	55 (10.2)	69 (10.6)	63 (11.3)	50 (8.5)
**Income per person in the household per month, n (%)**					
	<€250 (US $325)	118 (21.9)	139 (21.2)	121 (21.7)	146 (24.9)
	€250-€499 (US $325-$649)	229 (42.7)	323 (49.4)	272 (48.8)	264 (45.1)
	€500-€649 (US $650-$844)	86 (16.0)	99 (15.2)	122 (14.2)	96 (16.3)
	>€750 (US $975)	104 (19.4)	93 (14.2)	64 (15.3)	80 (13.7)
**BMI categories, n (%)**					
	Normal or underweight (<25)	52 (9.7)	58 (8.9)	45 (8.1)	44 (7.5)
	Preobese (<30)	164 (30.5)	183 (28.0)	181 (32.5)	197 (33.6)
	Obese class 1 (<35)	200 (37.2)	227 (34.7)	175 (31.4)	195 (33.3)
	Obese class 2 (<40)	77 (14.3)	122 (18.7)	103 (18.5)	99 (16.9)
	Obese class 3 or 4 (≥40)	44 (8.2)	64 (9.8)	53 (9.5)	51 (8.7)
BMI (kg/m^2^), mean (SD)		31.6 (5.7)	32.4 (6.0)	32.1 (5.8)	32.1 (6.0)
Duration of diabetes (years), mean (SD)		8.4 (6.8)	8.2 (6.1)	8.9 (6.3)	8.6 (6.8)
**Diabetes treatment, n (%)**					
	Only lifestyle	40 (7.5)	60 (9.2)	26 (4.7)	53 (9.0)
	Oral	394 (73.4)	445 (68.0)	413 (74.1)	395 (67.4)
	Injectable (insulin or GLP-1^e^)	12 (2.2)	17 (2.6)	17 (3.1)	25 (4.3)
	Oral+injectable	85 (15.8)	114 (17.4)	98 (17.6)	98 (16.7)
	Do not know/not answered	6 (1.1)	18 (2.8)	3 (0.5)	15 (2.6)
**HbA_1c_^f^ categories, n (%)**					
	<7%	258 (48.0)	351 (53.7)	241 (43.3)	304 (51.9)
	7.0%-8.0%	146 (27.2)	165 (25.2)	165 (29.6)	141 (24.1)
	8.1%-9.0%	66 (12.3)	75 (11.5)	82 (14.7)	67 (11.4)
	>9.0%	67 (12.5)	63 (9.6)	69 (12.4)	74 (12.6)
HbA_1c_ (%), mean (SD)		7.3 (1.5)	7.2 (1.4)	7.4 (1.5)	7.3 (1.5)
**Comorbidities, n (%)**					
	Hypertension	323 (60.3)	434 (66.4)	363 (65.2)	382 (67.1)
	Hypercholesterolemia	353 (65.7)	448 (68.0)	349 (62.7)	367 (64.0)
	Coronary artery disease	32 (6.0)	39 (6.0)	26 (4.67)	27 (3.9)
	Ictus	12 (2.2)	5 (0.8)	13 (2.3)	14 (2.1)
	Thyroid gland disorders	68 (12.7)	76 (11.6)	57 (9.7)	57 (11.8)

^a^PTI: intervention only for patients and family members.

^b^PFI: intervention only for health care professionals at primary care.

^c^CBI: combined intervention for patients and professionals.

^d^UC: usual care or control group.

^e^GLP-1: glucagon-like peptide-1.

^f^HbA_1c_: glycated hemoglobin.

The rate of attendance at educational sessions is also shown in Figure 2. The mean number of sessions attended by patients in the intervention for patients and combined intervention groups was 4.3 (SD 2.7) and 4.2 (SD 2.8), respectively. Overall, 87.5% (470/537) of the patients assigned to the intervention for patients group attended the first of the 8 educational sessions, which decreased to 59.2% (318/537) in the second session and to 45.8% (246/537) in the last session. In the combined intervention group, attendance rates were 73.4% (409/557), 57.8% (322/557), and 47.2% (263/557), respectively. All patients in the intervention groups received SMS during the 2 years of follow-up and had access to the web platform that contained the video recordings of all group sessions in addition to other educational materials. The average number of web-based questionnaires filled in by each patient was 16.3 (SD 29.4) in the intervention for patients group and 9.9 (SD 23.1) in the combined intervention group. These differences were statistically significant (*P*<.001) at the 2-year follow-up.

### Primary End Point: HbA1c

Multimedia Appendix 4 shows the adjusted differences in the mean HbA_1c_ levels at each follow-up evaluation and the adjusted differences in AUCs of HbA_1c_ throughout the whole study for each intervention group, in comparison with the usual care group. Compared with usual care, intervention for patients achieved a significant mean HbA_1c_ reduction of ‒0.27 (95% CI ‒0.45 to ‒0.10) at month 3 and ‒0.26 (95% CI ‒0.44 to ‒0.08) at month 6. Differences between intervention for patients and usual care groups were marginally significant at 12 months (*P*=.07). There were no statistically significant differences in mean HbA_1c_ levels in the intervention for professionals and combined intervention groups, when compared with the usual care group. With regard to the AUC of HbA_1c_, the effect of intervention for patients was marginally significant compared with usual care (*P*=.06), considering all the follow-up sessions.

The mean levels of HbA_1c_ across the study and their adjusted differences with regard to baseline values are shown in Multimedia Appendix 5 by the study arm. Mean HbA_1c_ levels of the intervention for patients group significantly improved during the first 12 months of follow-up, showing a maximal reduction at month 3 (‒0.35; 95% CI ‒0.48 to ‒0.22). The differences gradually diminished over time until they disappeared at months 18 and 24.

At month 3, a clinically relevant reduction in HbA_1c_ (at least 0.4%) was observed in 38.6% (207/537) of participants in the intervention for patients group and only in 20.3% (119/586) of patients with usual care (*P*<.001; Multimedia Appendix 6). Differences between both groups in the proportion of subjects with a clinically significant decrease in HbA_1c_ remained statistically significant until month 12 (191/537, 35.6% vs 152/586, 26.0%; *P*=.006) and marginally significant until month 18. The percentage of patients with clinically relevant decrease in HbA_1c_ was also significantly greater in the combined intervention group than in the usual care group at months 3, 6, and 18.

The results of the interventions were also analyzed in the relevant subgroup of uncontrolled patients with baseline HbA_1c_ >7%. As shown in Multimedia Appendix 7, for this subgroup, the differences in the HbA_1c_ reduction between the intervention for patients and usual care groups were statistically significant, favoring the intervention for patients group from months 3 to 12. The differences in HbA_1c_ AUC between the intervention groups and the usual care group considering the entire period were statistically significant for the intervention for patients and combined intervention: ‒0.26 (95% CI ‒0.48 to ‒0.04) and ‒0.25 (95% CI ‒0.47 to ‒0.03), respectively. For the intervention for professionals group, the differences were marginally statistically significant (*P*=.09).

All interventions led to a significant reduction in HbA_1c_ among subjects with baseline HbA_1c_ levels >7% across the entire study period (Multimedia Appendix 8). The differences at 24 months were ‒0.49 (95% CI ‒0.70 to ‒0.27) for intervention for patients, ‒0.35 (95% CI ‒0.59 to ‒0.14) for intervention for professionals, and ‒0.35 (95% CI ‒0.57 to ‒0.13) for combined intervention (Multimedia Appendix 8). Patients with usual care showed significant decreases in HbA_1c_ at months 12, 18, and 24.

Finally, in the subgroup with baseline HbA_1c_ levels >7%, the proportion of subjects with clinically significant reductions in HbA_1c_ (≥0.4%) was greater in the intervention for patients group than in the usual care group until month 12 (140/263, 53.1% vs 116/269, 43.2%; *P*=.049). The differences between the combined intervention and the usual care groups were significant at month 3 (Multimedia Appendix 6).

### Secondary End Points

Compared with usual care, the intervention for professionals group had significantly lower SBP at months 3 and 18 and the combined intervention group had significantly lower SBP at month 24 (Multimedia Appendix 4). Compared with their respective baseline values, mean SBP fell significantly in all study groups, but the difference was greatest for the combined intervention group at 24 months (‒7.5 mm Hg; 95% CI ‒9.8 to ‒5.2; Multimedia Appendix 5). For DBP, compared with usual care, we found significant reductions at months 3 and 24 for intervention for professionals and at months 12 and 24 for combined intervention (Multimedia Appendix 4). When compared with baseline, all groups improved; the maximum reduction was at 24 months for the combined intervention group, with a fall of ‒6.7 mm Hg (95% CI ‒8.2 to ‒5.3; Multimedia Appendix 5). The intervention for patients did not lead to a significant decrease in blood pressure compared with usual care (Multimedia Appendix 4).

Comparisons in BMI between the intervention for patients and usual care groups only attained statistically significant differences at month 3. None of the other interventions achieved greater BMI reductions than those observed for usual care (Multimedia Appendix 4). Compared with the baseline values, the mean values of BMI decreased in the intervention for professionals group throughout the follow-up and in the usual care group at months 3 and 24. The intervention for patients group experienced the greatest improvement and showed a statistically significant reduction at month 24: ‒0.78 kg/m^2^ (95% CI ‒1.0 to ‒0.6; Multimedia Appendix 5).

Multimedia Appendices 4 and 5 contain detailed biochemical, clinical, and anthropometric data for the whole sample. Multimedia Appendices 7 and 8 contain these data for the subgroup with basal HbA_1c_ >7%.

All 4 groups showed statistically significant improvements in total and LDL cholesterol levels at the end of follow-up. The differences between the intervention and usual care groups were not statistically significant. HDL cholesterol and triglyceride levels did not reveal clinically relevant changes.

We did not detect statistically significant differences in the comparison of intervention for patients and intervention for professionals groups in relation to the most intensive intervention in the combined intervention group regarding the primary or secondary outcomes in the AUC over the follow-up period, except for BMI, which had a difference in area of −0.29 (95% CI −0.57 to 0.01) kg/m^2^ in favor of the intervention for patients group.

For most clinical results, ICC values were low in every PHCP. Variance homogeneity was verified and thus reflected a very small effect associated with PHCP for intervention and control groups (similar clinical results among PHCP in every study arm). The ICC at the patient level was broad, accounting for considerable variations among individuals. Considering both ICC values, the results from the INDICA study appear to have good external validity.

## Discussion

### Principal Findings

The INDICA study assessed the effectiveness of multicomponent interventions to support decision making for the main actors involved in the management of T2DM (patients, relatives, and primary health care professionals) in many PHCPs [13]. We hypothesized that combining conventional educational activities with different ICT-based decision support tools would improve HbA_1c_ at long term (24 months) compared with usual care.

This study revealed that the intervention for patients group achieved a significant but temporary reduction of HbA_1c_, compared with the usual care group, which lasted for 6 months, with a gradual dilution effect from then onward. Interventions focused on health care professionals and on both patients and health care professionals did not translate into a significant lowering of HbA_1c_, in comparison with usual care, when evaluated in the whole study population. Even so, more than 30% of the participants belonging to the intervention for patients and combined intervention groups attained statistically and clinically relevant reductions in HbA_1c_ (>0.4%). These percentages were significantly greater than those observed in the control group at 12 months (for the intervention for patients group) and 18 months (for the combined intervention group).

It must be noted that, with the intention of assessing the effectiveness of the intervention for all patients with T2DM, the INDICA study did not limit inclusion of participants by their HbA_1c_ level. Therefore, the study’s power to find clinically relevant differences for the main outcome measures could have been insufficient, according to Jackson et al [23], as only 50.6% (1180/2334) of all participants had baseline HbA_1c_ concentrations >7% (mean 7.3%, SD 1.5). Nonetheless, the study’s sample size provided statistical power to examine the results of patients with worse metabolic control, allowing the comparison with other studies that limited recruitment to patients with poor metabolic control.

As expected, the magnitude and duration of the intervention effect was greater among patients with baseline HbA_1c_ >7%, mainly for the intervention for patients group, which showed a statistically significant reduction in HbA_1c_, in comparison with usual care, although the difference disappeared at 18 months. Moreover, considering the differences in the AUC values of HbA_1c_, our results provide evidence of effectiveness for both the intervention for patients and the combined intervention throughout the study period. These results support previous findings reporting greater effects for interventions on patients with higher baseline HbA_1c_ levels [24,25]. Similarly, the effectiveness of quality improvement strategies exclusively focused on health care providers seems to be beneficial only among patients with HbA_1c_ levels >8% [26].

The Mobile Diabetes Intervention Study (MDIS) published by Quinn et al [27] also reported a higher reduction in HbA_1c_ over 1 year among patients with T2DM (with baseline HbA_1c_=9.1%) by means of a multicomponent behavioral intervention exclusively for patients, without detecting effects on other relevant outcomes such as blood pressure or lipid levels.

Although MDIS provided evidence of sustained 12-month treatment difference in HbA_1c_, rather than *regression to the mean*, the INDICA results, for the whole sample, show a progressive effect reduction close to the baseline HbA_1c_ values. Similar to MDIS, the observed reduction in HbA_1c_ in the INDICA subgroup with baseline HbA_1c_>7% remained stable over the long term. However, evidence of long-term effectiveness of these complex interventions is not well stated yet because of the reduced number of studies providing results at 12 months of follow-up and beyond [28,29].

Several systematic reviews found that interventions based on ICTs led to significant improvements of 4% to 5% in HbA_1c_ compared with usual care [12,28,30,31], with effect differences according to the type of ICT used (internet, automated SMS, and apps) [11,12,32]. In contrast, smaller effects than those reported in our study for the intervention for patients and combined intervention groups were published for individual and group education among patients with HbA_1c_ levels >8% [33,34].

Beyond the reported effects on HbA_1c_, we also found an improvement in blood pressure monitoring for patients included in the 2 groups with intervening health professionals. Long-term reductions compared with the baseline were observed in SBP and DBP, with statistically significant differences in relation to usual care. These combined effects on HbA_1c_, SBP, and DBP, together with the improvement observed for BMI, might contribute to enhanced cardiovascular risk [35,36], suggesting the overall value of these comprehensive approach strategies addressing multiple components and actors involved in T2DM management [37]. Although some outcomes, such as the improvement of blood pressure, might require the involvement of health care providers, others, such as the reduction in HbA_1c_, will depend largely on the patients’ intervention. Thus, our findings provide long-term evidence on the effectiveness of multicomponent interventions to empower patients and support clinical decision making to improve T2DM outcomes beyond that published by Taylor et al [29] in their systematic review for self-management interventions for patients with chronic conditions. The potential expected clinical benefits, associated with the overall metabolic and cardiovascular risk improvement provided by INDICA over 2 years, could be estimated in the longer-term follow-up on both microvascular and macrovascular complications and mortality [38].

### Conceptual Frameworks

The assessed interventions were informed by conceptual frameworks of behavioral change [14] and applied to a large and heterogeneous sample of patients, caregivers, and professionals. The INDICA intervention characteristics were planned to increase the validity of the obtained data and the transferability of the interventions assessed. The key determinants of behavior change suggested by Michie et al [14] were considered for the INDICA interventions, with a higher degree of adherence in their design and implementation in the case of interventions for patients than for professionals, which could help explain the magnitude of the effect observed for HbA_1c_ among intervention groups. Furthermore, time constraints, staff turnover, and self-perception of work overload among health professionals limited the possibility of going deeper into the following dimensions: professional role, motivation and goals, social and professional influences, emotions, and action planning. A detailed description of the complex behavior change interventions applied was reported elsewhere [13] to promote replication at other sites. Other potential explanations for the unexpected differences between the intervention for patients and combined intervention groups were the higher attendance rate of patient and family members in the educational group sessions and a significantly higher rate of web questionnaire completion observed in the intervention for patients group. This higher rate of questionnaire completion was key to adjusting the individualized components of SMS messages, providing an extended exposure to web-based educational material. The high turnover among health care professionals in most PHCPs included in the study, as occurs in the real world, could also account for the lesser effect of the intervention for professionals and the combined intervention.

To maximize effectiveness, the INDICA interventions incorporated all the components of a technology-enabled self-management feedback cycle, connecting patients and the research team by using bidirectional communication, analyzing patient-provided behavior and health data, tailoring education, and personalizing feedback according to the eHealth Enhanced Chronic Care Model [24,39,40].

### Strengths and Limitations

This study has some limitations. First, it was difficult to obtain a full data set because of the high number of control visits and the duration of follow-up for many patients. Robust imputation techniques [19] were used to minimize the impact of missing data. Second, as previously mentioned, the high turnover among primary care professionals included in the study could explain the smaller than expected impact of the intervention for professionals and the combined intervention. Third, the fact that around 49.4% (1154/2334) of the whole patient sample had baseline HbA_1c_ <7% and only around 23.0% (536/2334) had basal HbA_1c_ levels ≥8% clearly limits the ability of interventions to reduce HbA_1c_. Fortunately, the available sample size was sufficient to find valid evidence. Fourth, similar to other reported findings [23,26,41], our usual care group was not a proper control group; it was subject to repetitive and intensive follow-up activities, including 6 different follow-up visits over the study to apply all prespecified questionnaires, in addition to clinical and laboratory tests. This intense follow-up activity could act as an intervention in itself, as patients might focus on important topics on which they had to pay attention. Fifth, INDICA interventions were not fully theory-based, making it more difficult to understand as to what works across contexts, populations, and behaviors. Finally, the INDICA study was not designed to test the efficacy of every component of the complex interventions assessed.

The strengths of the INDICA study include the pragmatic character of the trial and its wide sample size; the random assignment by clusters; the engagement, as research subjects, of all actors involved in management decisions; and the follow-up duration. Moreover, all educational group sessions and coaching activities by SMS were recorded to monitor and assess homogeneity, educator fidelity to interventions, and quality delivery. Educational workshops and periodic feedback to health care professionals were equally delivered to all participants in the intervention for professionals and the combined intervention.

The INDICA findings highlight the importance of conducting trials with long follow-up periods and sufficient statistical power to assess interventions of limited expected effect sizes but of high potential efficiency. ICT-supported interventions enable its extended and continuous usage by thousands of people in need to complement and spread interventions beyond the limited capacity of the health care systems to deliver usual care. We should be careful, however, to generalize the findings of INDICA. Interventions took place through PHCPs and were largely implemented through electronic communications. Health and digital literacy levels of the assessed population might vary with regard to other settings. Moreover, health care professionals were subject to differences in workload, interest and training in ICT used to support patients, access to CPG, and specialist support.

The potential effects of all these factors on the different study arms were minimized by randomization.

### Future Research

Future research on the effectiveness of these complex interventions should be complemented by the analysis of patients’ self-reported outcomes and intervention cost-effectiveness to fully inform clinical and health policy decision making. The effectiveness of these interventions should also be assessed after longer follow-up periods to allow the measurement of relevant clinical (micro and macrovascular) outcomes, together with the assessment of potential longer-term reinforcement of the most cost-effective interventions in the short term. The use of real-world data will efficiently help to provide this valuable information. Effectiveness and cost-effectiveness assessment according to patients’ clinical risk and health literacy levels are also highly relevant. Additional evidence on cost-effectiveness and budget impact analysis is needed to support health policy decision making in cases of limited funding to support all assessed interventions.

Theory-based research on complex interventions to promote behavior change is also needed, rather than theory-inspired research, if we are to achieve a sound scientific basis for the development and reporting of such interventions. Comparative effectiveness assessment among components of complex interventions is also of interest, although it will require additional funding.

Finally, qualitative research is also needed to better understand the relationships between patient and professional characteristics, their engagement, and the observed results.

### Conclusions

We found that INDICA interventions improved long-term metabolic control in patients with T2DM with uncontrolled basal HbA_1c_ values compared with the usual care group. We also found moderate but clinically and statistically significant effects on blood pressure reduction, contributing to reduced overall cardiovascular risk. The increasing access to computers, internet, and mobile phones, together with improvements in digital literacy, regardless of social status, sex, and age, make these complex interventions appropriate instruments to improve patient empowerment in the continuous management of their chronic diseases by tailoring interventions to individual needs and extending patient support beyond the limited capacities of conventional office-based care.

## Appendix

Multimedia Appendix 1 Screenshots of patients’ website.

Multimedia Appendix 2 Representative screenshots of automated decision aid tool embedded into the electronic clinical record.

Multimedia Appendix 3 Multiple imputation model.

Multimedia Appendix 4 Adjusted difference in means and area under the curve of each group compared with the usual care group for the whole sample.

Multimedia Appendix 5 Adjusted means for each group and intragroup differences compared with the baseline measurement for the whole sample.

Multimedia Appendix 6 Patients with clinically relevant changes in glycated hemoglobin and comparison with the usual care group.

Multimedia Appendix 7 Adjusted difference in means and area under the curve of each group compared with the usual care group for patients with a baseline glycated hemoglobin level >7%.

Multimedia Appendix 8 Adjusted means for each group and intragroup differences compared with the baseline measurement for patients with a baseline glycated hemoglobin level >7 %.

Multimedia Appendix 9 CONSORT-EHEALTH checklist (V 1.6.1).

## Abbreviations

AUC: area under the curve
CPG: clinical practice guideline
DBP: diastolic blood pressure
ECR: electronic clinical record
FCU: family care unit
HbA1c: glycated hemoglobin
HDL: high-density lipoprotein
ICC: intraclass correlation coefficient
ICT: information and communications technology
LDL: low-density lipoprotein
MDIS: Mobile Diabetes Intervention Study
PHCP: primary health care practice
RCT: randomized controlled trial
SBP: systolic blood pressure
T2DM: type 2 diabetes mellitus
