# Supplementary figures and images for: Effectiveness of Internet-Based Multicomponent Interventions for Patients and Health Care Professionals to Improve Clinical Outcomes in Type 2 Diabetes Evaluated Through the INDICA Study: Multiarm Cluster Randomized Controlled Trial
Source: JMIR Mhealth Uhealth. 2020 Nov 2;8(11):e18922. doi: 10.2196/18922 (PMC7669446; doi:10.2196/18922)

Multimedia Appendix 1. Screenshots of patients’ website


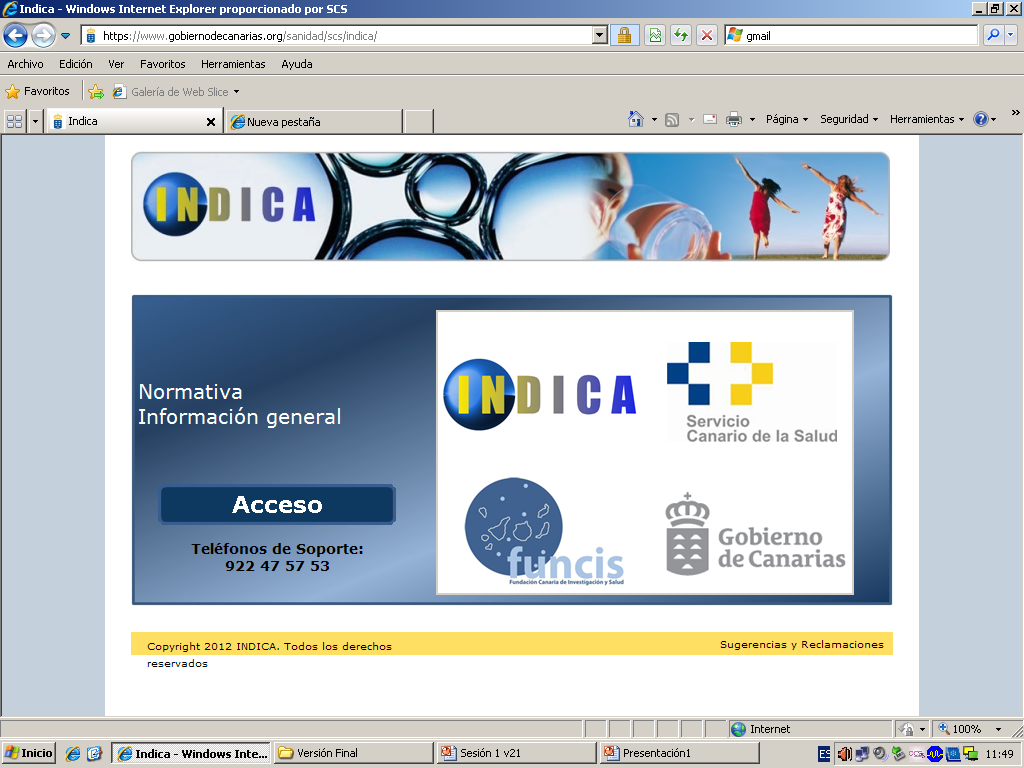


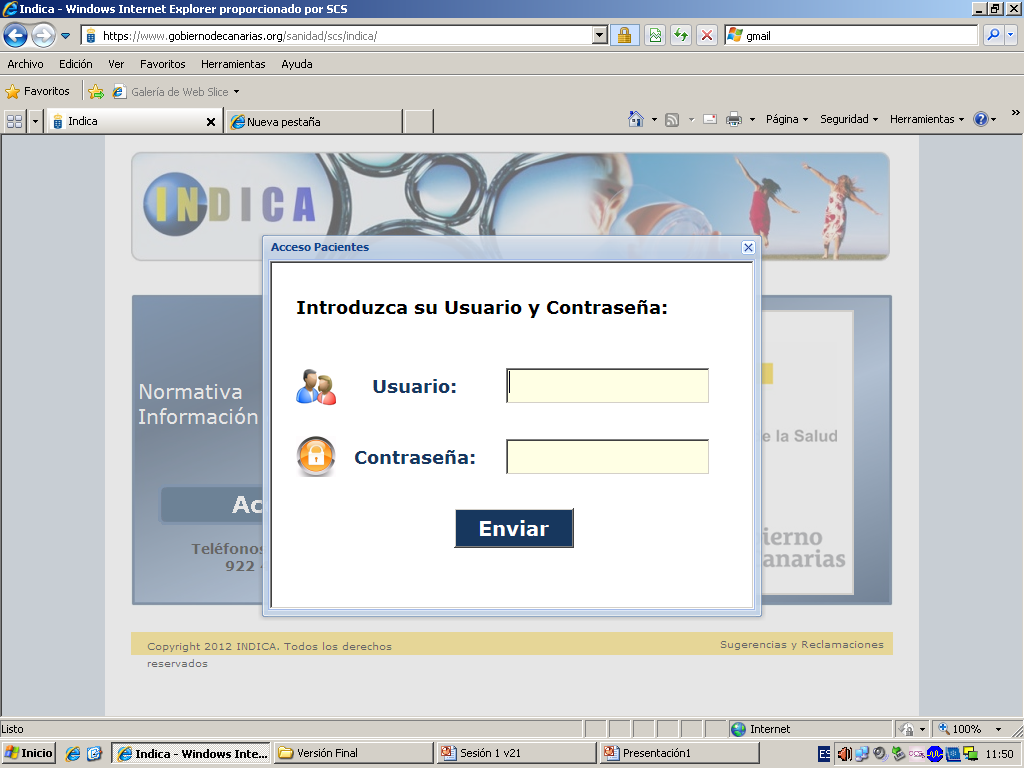


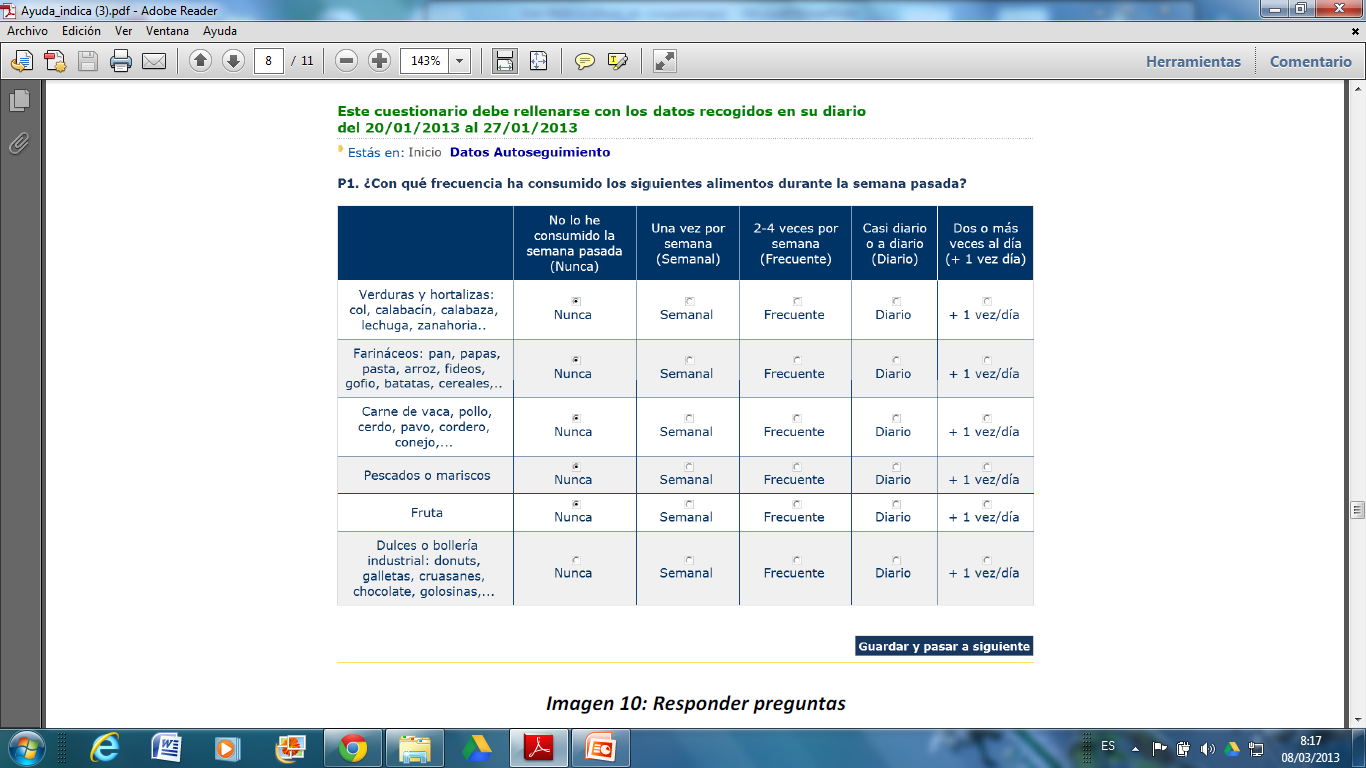

Supplement: Multimedia Appendix 1 [file mhealth_v8i11e18922_app1.doc]
