# Supplementary material for: Effectiveness of Internet-Based Multicomponent Interventions for Patients and Health Care Professionals to Improve Clinical Outcomes in Type 2 Diabetes Evaluated Through the INDICA Study: Multiarm Cluster Randomized Controlled Trial
Source: JMIR Mhealth Uhealth. 2020 Nov 2;8(11):e18922. doi: 10.2196/18922 (PMC7669446; doi:10.2196/18922)

Multimedia Appendix 2. Representative screenshots of automated decision aid tool embedded into the electronic clinical record.


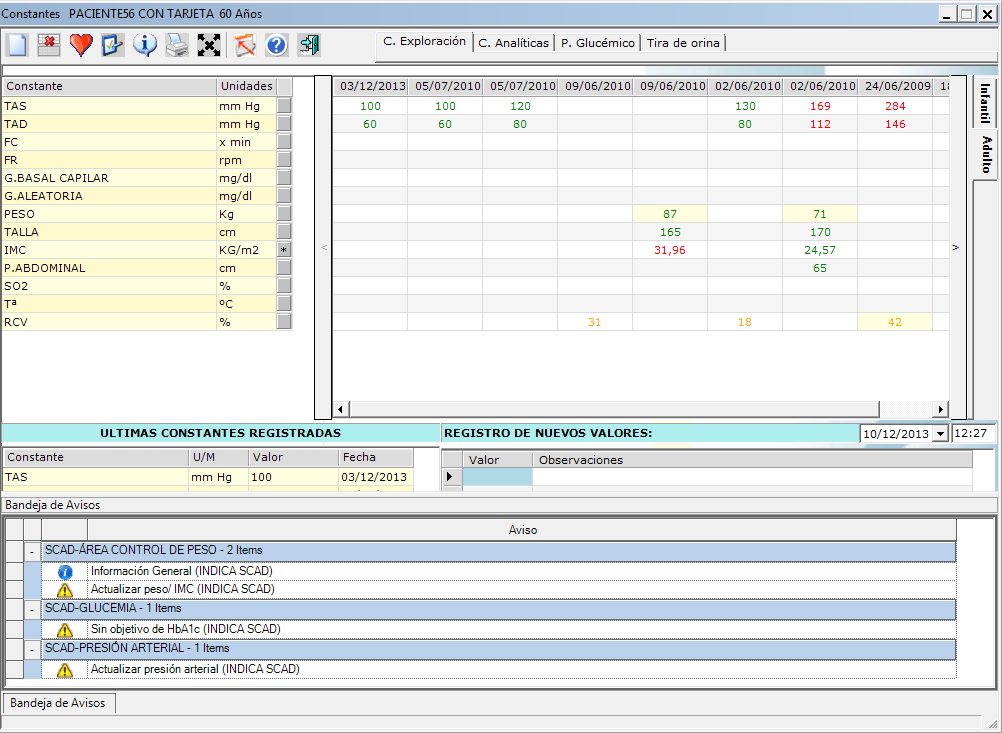


Message that include the evaluation of the patient’s health and recommendations based on the specific needs of every patient.


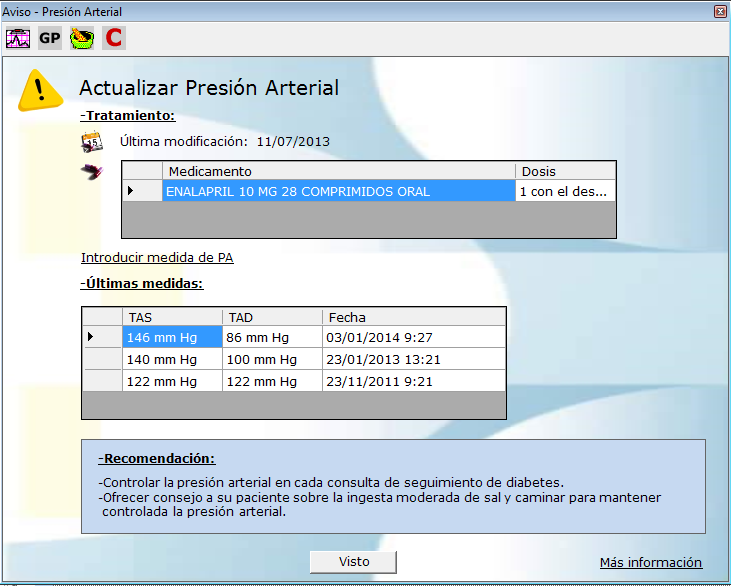


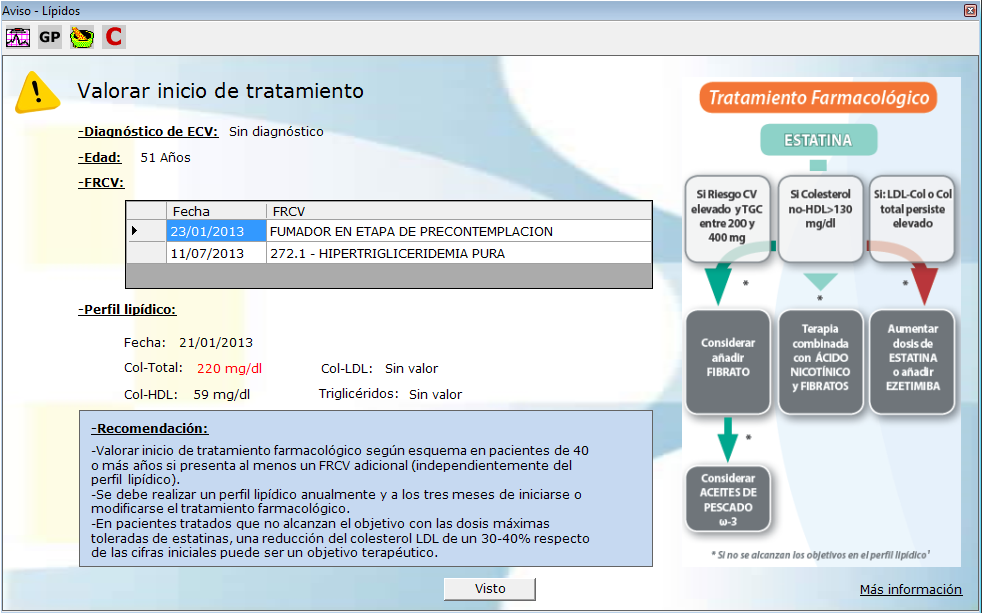


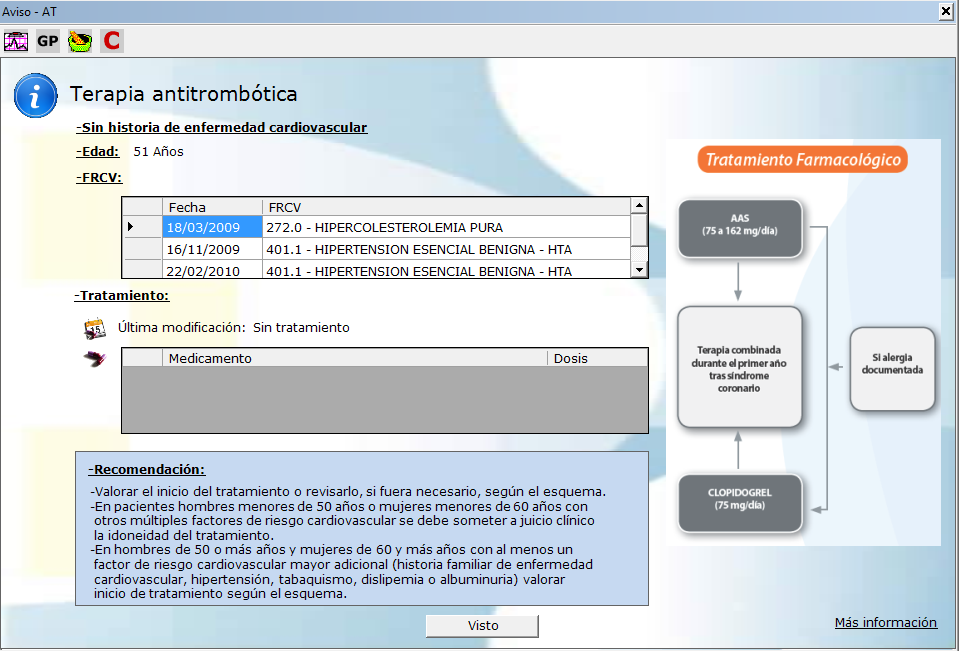

Supplement: Multimedia Appendix 2 [file mhealth_v8i11e18922_app2.doc]
