# Supplementary material for: Effectiveness of Internet-Based Multicomponent Interventions for Patients and Health Care Professionals to Improve Clinical Outcomes in Type 2 Diabetes Evaluated Through the INDICA Study: Multiarm Cluster Randomized Controlled Trial
Source: JMIR Mhealth Uhealth. 2020 Nov 2;8(11):e18922. doi: 10.2196/18922 (PMC7669446; doi:10.2196/18922)
Supplement: Multimedia Appendix 3 [file mhealth_v8i11e18922_app3.doc]

Multimedia Appendix 3. Multiple imputation model.

# Description of Missing data

# Percentage of missing data in primary endpoint, across observations

The percentage of missing data is maximum at 18 months, and decline a little at 24 months. The percentage is always less than 40%. There are a 4.5% (105/2,334) of patients with missing data in all the observations at follow-up (except in baseline).

|  | n (%) |
| --- | --- |
| **HbA1c, baseline** |  |
| Non missing data | 2250 (96.4) |
| Missing data | 84 (3.6) |
| **HbA1c, 3 months** |  |
| Non missing data | 1794 (76.9) |
| Missing data | 540 (23.1) |
| **HbA1c, 6 months** |  |
| Non missing data | 1641 (70.3) |
| Missing data | 693 (29.7) |
| **HbA1c, 12 months** |  |
| Non missing data | 1662 (71.2) |
| Missing data | 672 (28.8) |
| **HbA1c, 18 months** |  |
| Non missing data | 1562 (66.9) |
| Missing data | 772 (33.1) |
| **HbA1c, 24 months** |  |
| Non missing data | 1590 (68.1) |
| Missing data | 744 (31.9) |

#

# Mechanisms causing missing data

Rubin [1] classified missing data problems into three categories. In his theory every data point has some likelihood of being missing. The process that governs these probabilities is called the missing data mechanism or response mechanism.

There are three typical mechanism causing missing data: missing completely at random (**MCAR**), missing at random (**MAR**) and missing not at random (**MNAR**).

The data are said to be MCAR if the probability of being missing data depends only on the overall probability of being missing: the probability is the same for all cases and is unrelated to the data. When the mechanism is MCAR this causes enlarged standard errors due the reduced sample size, but does not cause bias. In this situation we can analyze the incomplete datasets. While it would be convenient, MCAR is often unrealistic. If the mechanism of missing data depends on observed data, we would have to reject MCAR and if we analyze the incomplete sample we would be assuming important bias effects.

The data are MAR if the missingness probability depends on observed information, including any design factors. If only depends on the observed data, then the missing data are missing at random given the observed data. MAR is more realistic than MCAR. In the presence of MAR, methods such as multiple imputation of full information direct maximum likelihood may lead to unbiased results. But nevertheless, methods such as listwise delection, mean imputation or last observation carried forward should be avoided.

Finally, the data are MNAR if the missingness probability depends on unobserved information, including the value missing itself. MNAR implies that the probability of being missing varies for reason that are unknown to us. MNAR is difficult to detect because in practice it´s impossible discard the existence of MNAR, since we would not have observations related with missing data.

# Evaluating if missing data is MCAR in INDICA dataset.

The mechanism is not MCAR if the missing data depending on observed variables. For data to be missing completely at random, the probability that Xi is missing is unrelated to the value of other variables in the analysis.

So, we checked if the probabily of missing observations depends on observable variables:

#### 1) Hba1c across observations depending on missing data at baseline for Hba1c.

Those patients that not have HbA1c at baseline, have worse levels at month 24 (*P=.*03). In the rest of the follow-ups no differences are find.

| **Variables** | **Non missing data at baseline (N=2250)** | **Missing data at baseline (N=84)** | ***P*** |
| --- | --- | --- | --- |
|  | Mean (SD) | Mean (SD) |
| **HbA1c, 3 months** (N1=1721 y N2=73) | 7.11 (1.33) | 7.3 (1.33) | .24 |
| **HbA1c, 6 months** (N1=1590 y N2=51) | 7.17 (1.33) | 7.45 (1.25) | .13 |
| **HbA1c, 12 months** (N1=1610 y N2=52) | 7.24 (1.39) | 7.26 (1.30) | .90 |
| **HbA1c, 18 months** (N1=1510 y N2=52) | 7.28 (1.39) | 7.74 (1.81) | .08 |
| **HbA1c, 24 months** (N1=1534 y N2=56) | 7.29 (1.36) | 7.68 (1.28) | .03 |
| N1: total number of patients with non missing data at each variable and with non missing data at baseline. N2: total number of patients with non missing data at each variable and with missing data at baseline. | | | |

#### 2) Hba1c across observations depending on missing data at month 24 for Hba1c.

Those patients that not have HbA1c at month 24, have worse levels at baseline (*P=*.006), month 3 (*P=*.02), month 12 (*P=*.016) and month 24 (*P=*.007).

| **Variables** | **Non missing data at month 24 (N=1590)** | **Missing data at month 24 (N=744)** | ***P*** |
| --- | --- | --- | --- |
|  | Mean (SD) | Mean (SD) |
| **HbA1c, baseline** (N1=1534 y N2=716) | 7.21 (1.4) | 7.41 (1.61) | .006 |
| **HbA1c, 3 months** (N1=1313 y N2=481) | 7.07 (1.28) | 7.25 (1.43) | .02 |
| **HbA1c, 6 months** (N1=1262 y N2=379) | 7.18 (1.32) | 7.17 (1.36) | .91 |
| **HbA1c, 12 months** (N1=1292 y N2=370) | 7.19 (1.33) | 7.4 (1.54) | .016 |
| **HbA1c, 18 months** (N1=1238 y N2=324) | 7.24 (1.35) | 7.51 (1.6) | .007 |

N1: total number of patients with non missing data at each variable and with non missing data at month 24. N2: total number of patients with non missing data at each variable and with missing data at month 24.

#### 3) Missing data across Hba1c depending on age.

Those patients with missing data in Hba1c are younger that patients with non missing data at baseline and 24 months.

| **Variables** | **Non missing data at baseline (N=2250)** | **Missing data at baseline (N=84)** | ***P*** |
| --- | --- | --- | --- |
| **Age, mean (SD)** | 55.81 (7.07) | 52.69 (8.24) | <.001 |
|  | **Non missing at month 24 (N=1590)** | **Missing at month 24 (N=744)** |  |
| **Age, mean (SD)** | 56.09 (6.82) | 54.86 (7.73) | <.001 |

### 4) Missing data across Hba1c depending on smoker at baseline.

Smokers at baseline have more missing data in Hba1c at month 6, 12, 18 and 24.

| **Variables** | **Non missing data at baseline (N=2250)** | **Missing data at baseline (N=84)** | ***P*** |
| --- | --- | --- | --- |
|  | n(%) | n(%) |  |
| **Smoker Vs Non smoker at baseline** | | | .077 |
| Non smoker, N=1810 | 1752(96.8) | 58(3.2) |  |
| Smoker, N=524 | 498(95) | 26(5) |  |
|  | **Non missing data at month 3 (N=1794)** | **Missing data at month 3 (N=540)** |  |
|  | n(%) | n(%) |  |
| **Smoker Vs Non smoker at month 3** | | | .227 |
| Non smoker, N=1810 | 1402(77.5) | 408(22.5) |  |
| Smoker, N=524 | 392(74.8) | 132(25.2) |  |
|  | **Non missing data at month 6 (N=1641)** | **Missing data at month 6 (N=693)** |  |
|  | n(%) | n(%) |  |
| **Smoker Vs Non smoker at month 6** | | | <.001 |
| Non smoker, N=1810 | 1312(72.5) | 498(27.5) |  |
| Smoker, N=524 | 329(62.8) | 195(37.2) |  |
|  | **Non missing data at month 12 (N=1662)** | **Missing data at month 12 (N=672)** |  |
|  | n(%) | n(%) |  |
| **Smoker Vs Non smoker at month 12** | | | <.001 |
| Non smoker, N=1810 | 1326(73.3) | 484(26.7) |  |
| Smoker, N=524 | 336(64.1) | 188(35.9) |  |
|  | **Non missing data at month 18 (N=1562)** | **Missing data at month 18 (N=772)** |  |
|  | n(%) | n(%) |  |
| **Smoker Vs Non smoker at month 18** | | | <.001 |
| Non smoker, N=1810 | 1259(69.6) | 551(30.4) |  |
| Smoker, N=524 | 303(57.8) | 221(42.2) |  |
|  | **Non missing data at month 24 (N=1590)** | **Missing data at month 24 (N=744)** |  |
|  | n(%) | n(%) |  |
| **Smoker Vs Non smoker at month 24** | | | <.001 |
| Non smoker, N=1810 | 1266(69.9) | 544(30.1) |  |
| Smoker, N=524 | 324(61.8) | 200(38.2) |  |

### 5) Missing data at Hba1c in month 24 depending on smoker at PHCP.

The percentage of missing data depends on PHCP in a 3.64% (Figure 1). There are some PHCP with almost 50% of missing data.

Figure 1. Percentage of missing data at 24 months in Hba1c.

##
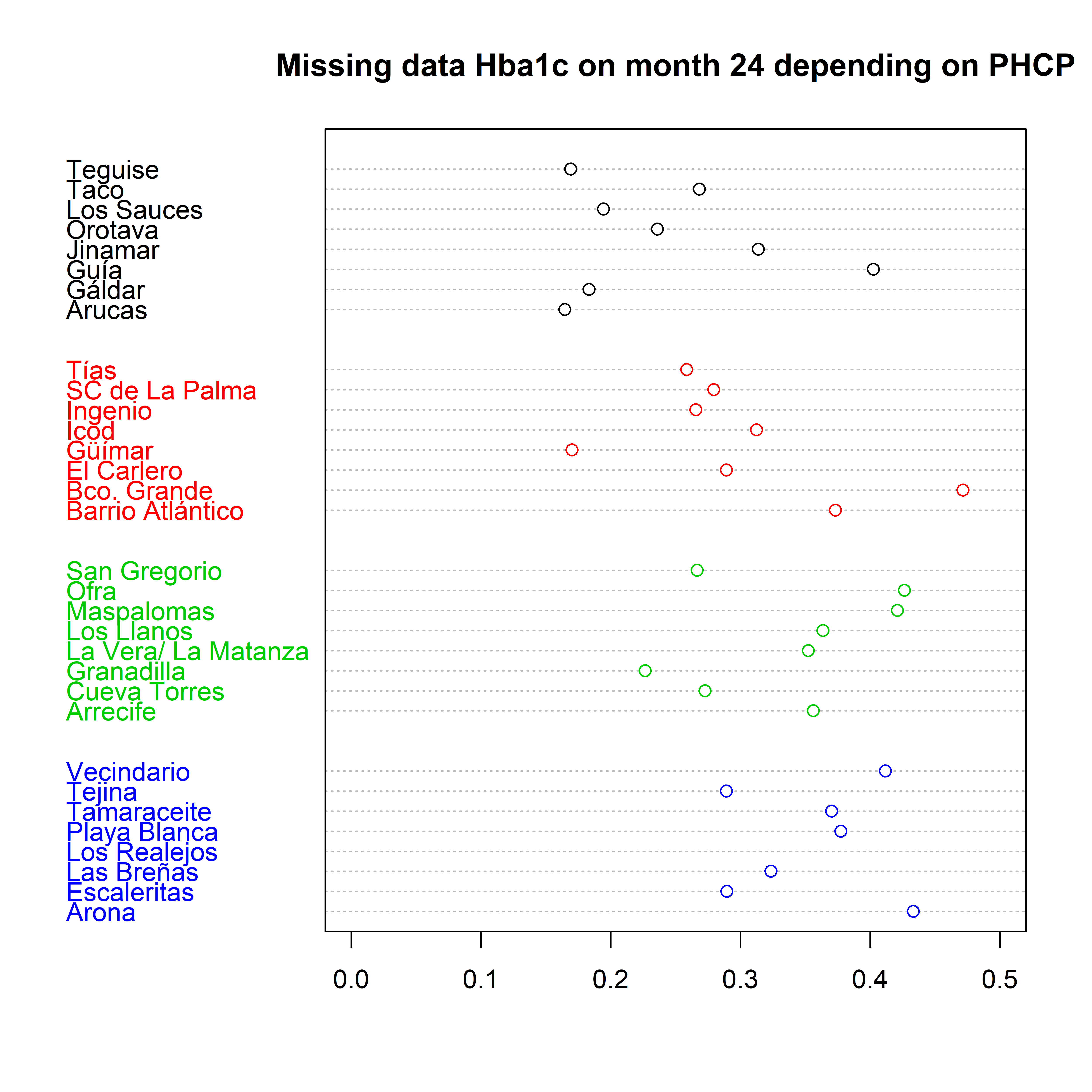


### 6) Hba1c at month 24 depending on several variables.

The missing datra of Hba1c at month 24 can be partially explained by a multilevel logistic model, using Hba1c baseline, age, smoker and sex as covariates.

|  | **OR** | **B** | **Std. Error** | **z value** | **Pr(>|z|)** |
| --- | --- | --- | --- | --- | --- |
| (Intercept) | 0.92 | -0.082 | 0.449 | -0.183 | .86 |
| HbA1c baseline | 1.0715 | 0.069 | 0.031 | 2.223 | .03 |
| Age, years | 0.978 | -0.022 | 0.007 | -3.382 | .001 |
| Smoker at baseline | 1.418 | 0.349 | 0.11 | 3.166 | .002 |
| Women | 0.869 | -0.14 | 0.094 | -1.481 | .14 |

ICC PHCP = 3.7%

**Conclusion about mechanism**

As our missing data are related to observable data, MCAR is not a mechanism plausible in our study. Since we have a lot of observed variables related with missing, the mechanism Missing not at Random (MNAR) is not plausible either. When the likelihood of missing data is related to observed variables, but not to unobserved variables, the missing data mechanism is referred to as missing at random (MAR) and this is the pattern in our missing data. Nevertheless, we cannot determinate if the mechanism is MNAR, because by definition the missing data are unknown and it can therefore not be assessed if the observed data can predict the unknown data.

When the likelihood of missing data is related to observed variables, but not to unobserved variables, the missing data mechanism is referred to as missing at random (MAR). Since we have a lot of observed variables related with missing, the mechanism Missing not at Random (MNAR) is not plausible. Nevertheless, we cannot determinate if the mechanism is MAR or MNAR, because by definition the missing data are unknown and it can therefore not be assessed if the observed data can predict the unknown data.

# Evaluating if missing data is monotone

The missing data is monotone if its columns can be reordered such that for any patient (a) if a data is missing all data after this value are also missing, and (b) if a data is observed all data before of this value are also are observed. In the presence of MAR, methods such as multiple imputation or full information direct maximum likelihood may lead to unbiased results. If missing data is not monotone, to avoid bias, a multiple imputation should be conducted using the chained equations or the MCMC method.

The missing data in INDICA is not Monotone because there are a total of 1159 patients with observations after at least one missing data. For this reason we used the chained equation method which is the most appropriate.

Figure 2. Monotone missing data


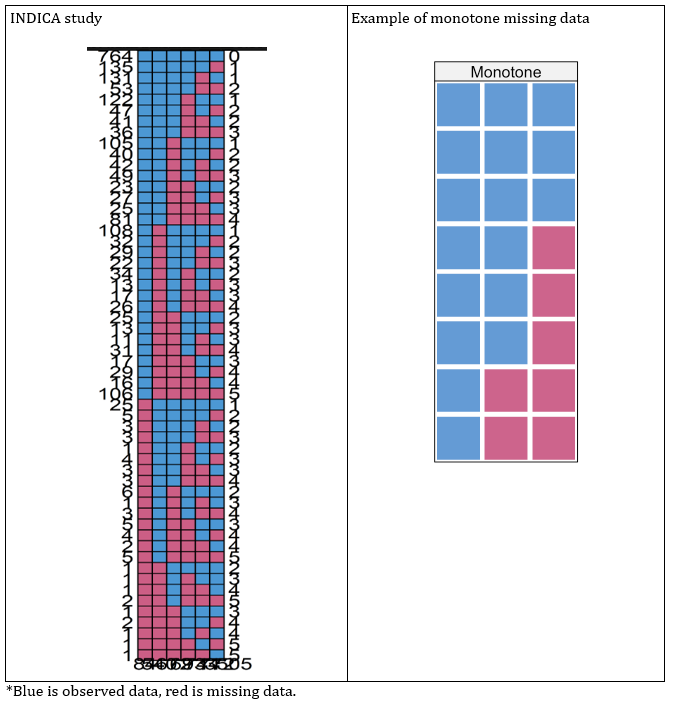


**References**

1. Rubin D. 1976. Inference and Missing Data. Biometrika 63 (3): 581–592. doi:10.2307/2335739

**Description of mechanism for imputation of missed data.**

Multiple imputation was performed by means of *mi impute chained* using the software Stata 15.0. Imputations were performed in a differentiated way for each of the four treatment groups. The following variables were considered regular and used as predictors to perform imputations: age of onset of the study, sex, baseline smoker status, oral anti-diabetics and basic health area. A total of 136 variables were imputed. Imputation of variables was organized starting from those that had less data lost (e.g. age of onset of the diabetes or level of studies). Each variable was imputed in chronological order: baseline first and afterwards 3, 6, 12, 18 and 24 months. As a general rule, the latest available information of the variable to impute was used. When information from other variables was used the information from the same time moment was used. The imputation was not performed using secondary variables as random effects without fixed effects being used. A total of 100 imputations was performed for every missed data. For some variables the variable ZBS was not used as predictor due to convergence problems because of problems of full separation in the logistic or ordinal models.

The following table shows the order of imputation of the variables, the variables used in the imputation, the prediction model and the number of lost data for this variable.

|  | **Imputed variable** | **Variables used in the imputation** | **Imputation Model** | **N missed** |
| --- | --- | --- | --- | --- |
| 1 | Duration of Diabetes (years) | PHCP, Age, Sex, Smoking status baseline, Diabetes treatment baseline | Pmm, knn(3) | 6 |
| 2 | Education | PHCP, Age, Sex, Smoking status baseline, Diabetes treatment baseline, Duration of Diabetes | Ologit | 24 |
| 3 | Laboral Status | PHCP, Age, Sex, Smoking status baseline, Diabetes treatment baseline, Duration of Diabetes | Logit | 43 |
| 4 | BMI, baseline | PHCP, Age, Sex, Smoking status baseline, Diabetes treatment baseline, Duration of Diabetes, Laboral Status baseline | Pmm, knn(3) | 5 |
| 5 | BMI, 3 months | PHCP, Age, Sex, Smoking status baseline, Diabetes treatment 3 months, Duration of Diabetes, Laboral Status baseline, BMI baseline | Pmm, knn(3) | 478 |
| 6 | BMI, 6 months | PHCP, Age, Sex, Smoking status baseline, Diabetes treatment 6 months, Duration of Diabetes, Laboral Status baseline, BMI 3 months | Pmm, knn(3) | 567 |
| 7 | BMI, 12 months | PHCP, Age, Sex, Smoking status baseline, Diabetes treatment 12 months, Duration of Diabetes, Laboral Status baseline, BMI 6 months | Pmm, knn(3) | 669 |
| 8 | BMI, 18 months | PHCP, Age, Sex, Smoking status baseline, Diabetes treatment 18 months, Duration of Diabetes, Laboral Status baseline, BMI 12 months | Pmm, knn(3) | 744 |
| 9 | BMI, 24 months | PHCP, Age, Sex, Smoking status baseline, Diabetes treatment 24 months, Duration of Diabetes, Laboral Status baseline, BMI 18 months | Pmm, knn(3) | 667 |
| 10 | Waist circumference, baseline | PHCP, Age, Sex, Smoking status baseline, Diabetes treatment baseline, Duration of Diabetes, Laboral Status baseline, BMI baseline | Pmm, knn(3) | 65 |
| 11 | Waist circumference, 3 months | PHCP, Age, Sex, Smoking status baseline, Diabetes treatment 3 months, Duration of Diabetes, Laboral Status baseline, BMI 3 months, Waist Circumference baseline | Pmm, knn(3) | 485 |
| 12 | Waist circumference, 6 months | PHCP, Age, Sex, Smoking status baseline, Diabetes treatment 6 months, Duration of Diabetes, Laboral Status baseline, BMI 6 months, Waist Circumference 3 months | Pmm, knn(3) | 574 |
| 13 | Waist circumference, 12 months | PHCP, Age, Sex, Smoking status baseline, Diabetes treatment 12 months, Duration of Diabetes, Laboral Status baseline, BMI 12 months, Waist Circumference 6 months | Pmm, knn(3) | 665 |
| 14 | Waist circumference, 18 months | PHCP, Age, Sex, Smoking status baseline, Diabetes treatment 18 months, Duration of Diabetes, Laboral Status baseline, BMI 18 months, Waist Circumference 12 months | Pmm, knn(3) | 747 |
| 15 | Waist circumference, 24 months | PHCP, Age, Sex, Smoking status baseline, Diabetes treatment 24 months, Duration of Diabetes, Laboral Status baseline, BMI 24 months, Waist Circumference 18 months | Pmm, knn(3) | 666 |
| 16 | Waist to hip ratio, Baseline | PHCP, Age, Sex, Smoking status baseline, Diabetes treatment baseline, Duration of Diabetes, Laboral Status baseline, BMI baseline, Waist Circumference baseline | Pmm, knn(3) | 66 |
| 17 | Waist to hip ratio, 3 months | PHCP, Age, Sex, Smoking status baseline, Diabetes treatment 3 months, Duration of Diabetes, Laboral Status baseline, BMI 3 months, waist circumference 3 months, Waist to Hip Ratio baseline | Pmm, knn(3) | 485 |
| 18 | Waist to hip ratio, 6 months | PHCP, Age, Sex, Smoking status baseline, Diabetes treatment 6 months, Duration of Diabetes, Laboral Status baseline, BMI 6 months, waist circumference 6 months, Waist to Hip Ratio 3 months | Pmm, knn(3) | 575 |
| 19 | Waist to hip ratio, 12 months | PHCP, Age, Sex, Smoking status baseline, Diabetes treatment 12 months, Duration of Diabetes, Laboral Status baseline, BMI 12 months, waist circumference 12 months, Waist to Hip Ratio 6 months | Pmm, knn(3) | 669 |
| 20 | Waist to hip ratio, 18 months | PHCP, Age, Sex, Smoking status baseline, Diabetes treatment 18 months, Duration of Diabetes, Laboral Status baseline, BMI 18 months, waist circumference 18 months, Waist to Hip Ratio 12 months | Pmm, knn(3) | 747 |
| 21 | Waist to hip ratio, 24 months | PHCP, Age, Sex, Smoking status baseline, Diabetes treatment 24 months, Duration of Diabetes, Laboral Status baseline, BMI 24 months, waist circumference 24 months, Waist to Hip Ratio 18 months | Pmm, knn(3) | 667 |
| 22 | Systolic blood pressure, baseline | PHCP, Age, Sex, Smoking status baseline, Diabetes treatment baseline, Duration of Diabetes, Laboral Status baseline, BMI baseline | Pmm, knn(3) | 68 |
| 23 | Systolic blood pressure, 3 months | PHCP, Age, Sex, Smoking status baseline, Diabetes treatment 3 months, Duration of Diabetes, Laboral Status baseline, BMI 3 months, SBP baseline | Pmm, knn(3) | 467 |
| 24 | Systolic blood pressure, 6 months | PHCP, Age, Sex, Smoking status baseline, Diabetes treatment 6 months, Duration of Diabetes, Laboral Status baseline, BMI 6 months, SBP 3 months | Pmm, knn(3) | 567 |
| 25 | Systolic blood pressure, 12 months | PHCP, Age, Sex, Smoking status baseline, Diabetes treatment 12 months, Duration of Diabetes, Laboral Status baseline, BMI 12 months, SBP 6 months | Pmm, knn(3) | 665 |
| 26 | Systolic blood pressure, 18 months | PHCP, Age, Sex, Smoking status baseline, Diabetes treatment 18 months, Duration of Diabetes, Laboral Status baseline, BMI 18 months, SBP 12 months | Pmm, knn(3) | 746 |
| 27 | Systolic blood pressure, 24 months | PHCP, Age, Sex, Smoking status baseline, Diabetes treatment 24 months, Duration of Diabetes, Laboral Status baseline, BMI 24 months, SBP 18 months | Pmm, knn(3) | 663 |
| 28 | Diastolic blood pressure, baseline | PHCP, Age, Sex, Smoking status baseline, Diabetes treatment baseline, Duration of Diabetes, Laboral Status baseline, BMI baseline, SBP baseline | Pmm, knn(3) | 68 |
| 29 | Diastolic blood pressure, 3 months | PHCP, Age, Sex, Smoking status baseline, Diabetes treatment 3 months, Duration of Diabetes, Laboral Status baseline, BMI 3 months, SBP 3 months, DBP baseline | Pmm, knn(3) | 467 |
| 30 | Diastolic blood pressure, 6 months | PHCP, Age, Sex, Smoking status baseline, Diabetes treatment 6 months, Duration of Diabetes, Laboral Status baseline, BMI 6 months, SBP 6 months, DBP 3 months | Pmm, knn(3) | 567 |
| 31 | Diastolic blood pressure, 12 months | PHCP, Age, Sex, Smoking status baseline, Diabetes treatment 12 months, Duration of Diabetes, Laboral Status baseline, BMI 12 months, SBP 12 months, DBP 6 months | Pmm, knn(3) | 665 |
| 32 | Diastolic blood pressure, 18 months | PHCP, Age, Sex, Smoking status baseline, Diabetes treatment 18 months, Duration of Diabetes, Laboral Status baseline, BMI 18 months, SBP 18 months, DBP 12 months | Pmm, knn(3) | 745 |
| 33 | Diastolic blood pressure, 24 months | PHCP, Age, Sex, Smoking status baseline, Diabetes treatment 24 months, Duration of Diabetes, Laboral Status baseline, BMI 24 months, SBP 24 months, DBP 18 months | Pmm, knn(3) | 663 |
| 34 | Hba1c, baseline | PHCP, Age, Sex, Smoking status baseline, Diabetes treatment baseline, Duration of Diabetes, Laboral Status baseline, Education baseline, BMI baseline, SBP baseline, Morisky Scale baseline, ADDQoL baseline | Pmm, knn(3) | 84 |
| 35 | Hba1C, 3 months | PHCP, Age, Sex, Smoking status 3 months, Diabetes treatment 3 months, Duration of Diabetes, Laboral Status baseline, Education baseline, BMI 3 months, SBP 3 months, Morisky Scale baseline, ADDQoL baseline, HbA1c baseline | Pmm, knn(3) | 538 |
| 36 | Hba1C, 6 months | PHCP, Age, Sex, Smoking status 6 months, Diabetes treatment 6 months, Duration of Diabetes, Laboral Status baseline, Education baseline, BMI 6 months, SBP 6 months, Morisky Scale 6 months, ADDQoL 6 months, HbA1c 3 months | Pmm, knn(3) | 689 |
| 37 | Hba1C, 12 months | PHCP, Age, Sex, Smoking status 12 months, Diabetes treatment 12 months, Duration of Diabetes, Laboral Status baseline, Education baseline, BMI 12 months, SBP 12 months, Morisky Scale 12 months, ADDQoL 12 months, HbA1c 6 months | Pmm, knn(3) | 665 |
| 38 | Hba1C, 18 months | PHCP, Age, Sex, Smoking status 18 months, Diabetes treatment 18 months, Duration of Diabetes, Laboral Status baseline, Education baseline, BMI 18 months, SBP 18 months, Morisky Scale 18 months, ADDQoL 18 months, HbA1c 12 months | Pmm, knn(3) | 768 |
| 39 | Hba1C, 24 months | PHCP, Age, Sex, Smoking status 24 months, Diabetes treatment 24 months, Duration of Diabetes, Laboral Status baseline, Education baseline, BMI 24 months, SBP 24 months, Morisky Scale 24 months, ADDQoL 24 months, HbA1c 18 months | Pmm, knn(3) | 737 |
| 40 | Fasting serum glucose, baseline | PHCP, Age, Sex, Smoking status baseline, Diabetes treatment baseline, Duration of Diabetes, Laboral Status baseline, Education baseline, BMI baseline, SBP baseline, Morisky Scale baseline, ADDQoL baseline, HbA1c baseline | Pmm, knn(3) | 44 |
| 41 | Fasting serum glucose, 6 months | PHCP, Age, Sex, Smoking status 6 months, Diabetes treatment 6 months, Duration of Diabetes, Laboral Status baseline, Education baseline, BMI 6 months, SBP 6 months, Morisky Scale 6 months, ADDQoL 6 months, HbA1c 6 months, Fasting serum glucose baseline | Pmm, knn(3) | 667 |
| 42 | Fasting serum glucose, 12 months | PHCP, Age, Sex, Smoking status 12 months, Diabetes treatment 12 months, Duration of Diabetes, Laboral Status baseline, Education baseline, BMI 12 months, SBP 12 months, Morisky Scale 12 months, ADDQoL 12 months, HbA1c 12 months, Fasting serum glucose 6 months | Pmm, knn(3) | 446 |
| 43 | Fasting serum glucose, 24 months | PHCP, Age, Sex, Smoking status 24 months, Diabetes treatment 24 months, Duration of Diabetes, Laboral Status baseline, Education baseline, BMI 24 months, SBP 24 months, Morisky Scale 24 months, ADDQoL 24 months, HbA1c 24 months, Fasting serum glucose 12 months | Pmm, knn(3) | 705 |
| 44 | Total cholesterol, baseline | PHCP, Age, Sex, Smoking status baseline, Diabetes treatment baseline, Duration of Diabetes, Laboral Status baseline, Education baseline, BMI baseline, SBP baseline, Morisky Scale baseline, ADDQoL baseline, HbA1c baseline, Fasting serum glucose baseline | Pmm, knn(3) | 46 |
| 45 | Total cholesterol, 6 months | PHCP, Age, Sex, Smoking status 6 months, Diabetes treatment 6 months, Duration of Diabetes, Laboral Status baseline, Education baseline, BMI 6 months, SBP 6 months, Morisky Scale 6 months, ADDQoL 6 months, HbA1c 6 months, Fasting serum glucose 6 months, Total colesterol baseline | Pmm, knn(3) | 676 |
| 46 | Total cholesterol, 12 months | PHCP, Age, Sex, Smoking status 12 months, Diabetes treatment 12 months, Duration of Diabetes, Laboral Status baseline, Education baseline, BMI 12 months, SBP 12 months, Morisky Scale 12 months, ADDQoL 12 months, HbA1c 12 months, Fasting serum glucose 12 months, Total colesterol 6 months | Pmm, knn(3) | 460 |
| 47 | Total cholesterol, 24 months | PHCP, Age, Sex, Smoking status 24 months, Diabetes treatment 24 months, Duration of Diabetes, Laboral Status baseline, Education baseline, BMI 24 months, SBP 24 months, Morisky Scale 24 months, ADDQoL 24 months, HbA1c 24 months, Fasting serum glucose 24 months, Total colesterol 12 months | Pmm, knn(3) | 705 |
| 48 | LDL, baseline | PHCP, Age, Sex, Smoking status baseline, Diabetes treatment baseline, Duration of Diabetes, Laboral Status baseline, Education baseline, BMI baseline, SBP baseline, Morisky Scale baseline, ADDQoL baseline, HbA1c baseline, Fasting serum glucose baseline, Total colesterol baseline | Pmm, knn(3) | 102 |
| 49 | LDL, 6 months | PHCP, Age, Sex, Smoking status 6 months, Diabetes treatment 6 months, Duration of Diabetes, Laboral Status baseline, Education baseline, BMI 6 months, SBP 6 months, Morisky Scale 6 months, ADDQoL 6 months, HbA1c 6 months, Fasting serum glucose 6 months, Total colesterol 6 months, LDL baseline | Pmm, knn(3) | 736 |
| 50 | LDL, 12 months | PHCP, Age, Sex, Smoking status 12 months, Diabetes treatment 12 months, Duration of Diabetes, Laboral Status baseline, Education baseline, BMI 12 months, SBP 12 months, Morisky Scale 12 months, ADDQoL 12 months, HbA1c 12 months, Fasting serum glucose 12 months, Total colesterol 12 months, LDL 6 months | Pmm, knn(3) | 550 |
| 51 | LDL, 24 months | PHCP, Age, Sex, Smoking status 24 months, Diabetes treatment 24 months, Duration of Diabetes, Laboral Status baseline, Education baseline, BMI 24 months, SBP 24 months, Morisky Scale 24 months, ADDQoL 24 months, HbA1c 24 months, Fasting serum glucose 24 months, Total colesterol 24 months, LDL 12 months | Pmm, knn(3) | 765 |
| 52 | HDL, baseline | PHCP, Age, Sex, Smoking status baseline, Diabetes treatment baseline, Duration of Diabetes, Laboral Status baseline, Education baseline, BMI baseline, SBP baseline, Morisky Scale baseline, ADDQoL baseline, HbA1c baseline, Fasting serum glucose baseline, Total colesterol baseline, LDL baseline, Waist to hip ratio baseline, Waist circumference baseline | Pmm, knn(3) | 55 |
| 53 | HDL, 6 months | PHCP, Age, Sex, Smoking status 6 months, Diabetes treatment 6 months, Duration of Diabetes, Laboral Status baseline, Education baseline, BMI 6 months, SBP 6 months, Morisky Scale 6 months, ADDQoL 6 months, HbA1c 6 months, Fasting serum glucose 6 months, Total colesterol 6 months, LDL 6 months, Waist to hip ratio 6 months, Waist circumference 6 months, HDL baseline | Pmm, knn(3) | 701 |
| 54 | HDL, 12 months | PHCP, Age, Sex, Smoking status 12 months, Diabetes treatment 12 months, Duration of Diabetes, Laboral Status baseline, Education baseline, BMI 12 months, SBP 12 months, Morisky Scale 12 months, ADDQoL 12 months, HbA1c 12 months, Fasting serum glucose 12 months, Total colesterol 12 months, LDL 12 months, Waist to hip ratio 12 months, Waist circumference 12 months, HDL 6 months | Pmm, knn(3) | 493 |
| 55 | HDL, 24 months | PHCP, Age, Sex, Smoking status 24 months, Diabetes treatment 24 months, Duration of Diabetes, Laboral Status baseline, Education baseline, BMI 24 months, SBP 24 months, Morisky Scale 24 months, ADDQoL 24 months, HbA1c 24 months, Fasting serum glucose 24 months, Total colesterol 24 months, LDL 24 months, Waist to hip ratio 24 months, Waist circumference 24 months, HDL 12 months | Pmm, knn(3) | 720 |
| 56 | Triglycerides, Baseline | PHCP, Age, Sex, Smoking status baseline, Diabetes treatment baseline, Duration of Diabetes, Laboral Status baseline, Education baseline, BMI baseline, SBP baseline, Morisky Scale baseline, ADDQoL baseline, HbA1c baseline, Fasting serum glucose baseline, Total colesterol baseline, LDL baseline, Waist to hip ratio baseline, Waist circumference baseline, HDL baseline | Pmm, knn(3) | 49 |
| 57 | Triglycerides, 6 months | PHCP, Age, Sex, Smoking status 6 months, Diabetes treatment 6 months, Duration of Diabetes, Laboral Status baseline, Education baseline, BMI 6 months, SBP 6 months, Morisky Scale 6 months, ADDQoL 6 months, HbA1c 6 months, Fasting serum glucose 6 months, Total colesterol 6 months, LDL 6 months, Waist to hip ratio 6 months, Waist circumference 6 months, HDL 6 months, Triglycerides baseline | Pmm, knn(3) | 681 |
| 58 | Triglycerides, 12 months | PHCP, Age, Sex, Smoking status 12 months, Diabetes treatment 12 months, Duration of Diabetes, Laboral Status baseline, Education baseline, BMI 12 months, SBP 12 months, Morisky Scale 12 months, ADDQoL 12 months, HbA1c 12 months, Fasting serum glucose 12 months, Total colesterol 12 months, LDL 12 months, Waist to hip ratio 12 months, Waist circumference 12 months, HDL 12 months, Triglycerides 6 months | Pmm, knn(3) | 461 |
| 59 | Triglycerides, 24 months | PHCP, Age, Sex, Smoking status 24 months, Diabetes treatment 24 months, Duration of Diabetes, Laboral Status baseline, Education baseline, BMI 24 months, SBP 24 months, Morisky Scale 24 months, ADDQoL 24 months, HbA1c 24 months, Fasting serum glucose 24 months, Total colesterol 24 months, LDL 24 months, Waist to hip ratio 24 months, Waist circumference 24 months, HDL 24 months, Triglycerides 12 months | Pmm, knn(3) | 700 |
| 60 | Serum Creatinine, baseline | PHCP, Age, Sex, Smoking status baseline, Diabetes treatment baseline, Duration of Diabetes, Laboral Status baseline, Education baseline, BMI baseline, SBP baseline, Morisky Scale baseline, ADDQoL baseline, HbA1c baseline, Fasting serum glucose baseline, Total colesterol baseline, LDL baseline, Triglycerides baseline | Pmm, knn(3) | 55 |
| 61 | Serum Creatinine, 12 months | PHCP, Age, Sex, Smoking status 12 months, Diabetes treatment 12 months, Duration of Diabetes, Laboral Status baseline, Education baseline, BMI 12 months, SBP 12 months, Morisky Scale 12 months, ADDQoL 12 months, HbA1c 12 months, Fasting serum glucose 12 months, Total colesterol 12 months, LDL 12 months, Triglycerides 12 months, Serum Creatinine baseline | Pmm, knn(3) | 467 |
| 62 | Serum Creatinine, 24 months | PHCP, Age, Sex, Smoking status 24 months, Diabetes treatment 24 months, Duration of Diabetes, Laboral Status baseline, Education baseline, BMI 24 months, SBP 24 months, Morisky Scale 24 months, ADDQoL 24 months, HbA1c 24 months, Fasting serum glucose 24 months, Total colesterol 24 months, LDL 24 months, Triglycerides 24 months, Serum Creatinine 12 months | Pmm, knn(3) | 699 |
| 63 | Glomerular filtration rate, Baseline | PHCP, Age, Sex, Smoking status baseline, Diabetes treatment baseline, Duration of Diabetes, Laboral Status baseline, Education baseline, BMI baseline, SBP baseline, Morisky Scale baseline, ADDQoL baseline, HbA1c baseline, Fasting serum glucose baseline, Total colesterol baseline, LDL baseline, Triglycerides baseline, Serum Creatinine baseline | Pmm, knn(3) | 55 |
| 64 | Glomerular filtration rate, 12 months | PHCP, Age, Sex, Smoking status 12 months, Diabetes treatment 12 months, Duration of Diabetes, Laboral Status baseline, Education baseline, BMI 12 months, SBP 12 months, Morisky Scale 12 months, ADDQoL 12 months, HbA1c 12 months, Fasting serum glucose 12 months, Total colesterol 12 months, LDL 12 months, Triglycerides 12 months, Serum Creatinine baseline, Glomerular filtration rate baseline | Pmm, knn(3) | 468 |
| 65 | Glomerular filtration rate, 24 months | PHCP, Age, Sex, Smoking status 24 months, Diabetes treatment 24 months, Duration of Diabetes, Laboral Status baseline, Education baseline, BMI 24 months, SBP 24 months, Morisky Scale 24 months, ADDQoL 24 months, HbA1c 24 months, Fasting serum glucose 24 months, Total colesterol 24 months, LDL 24 months, Triglycerides 24 months, Serum Creatinine 24 months, Glomerular filtration rate 12 months | Pmm, knn(3) | 705 |
