# Supplementary material for: Effectiveness of Internet-Based Multicomponent Interventions for Patients and Health Care Professionals to Improve Clinical Outcomes in Type 2 Diabetes Evaluated Through the INDICA Study: Multiarm Cluster Randomized Controlled Trial
Source: JMIR Mhealth Uhealth. 2020 Nov 2;8(11):e18922. doi: 10.2196/18922 (PMC7669446; doi:10.2196/18922)
Supplement: Multimedia Appendix 4 [file mhealth_v8i11e18922_app4.doc]

Multimedia Appendix 4. Adjusted difference in means and area under the curve of each group compared with the usual care group for the whole sample

|  | **Adjusted difference in means compared to the UC^a^ group: Mean (95%CI)** | | | | | | | | | | **Difference in AUC^b^ compared to the UC group (95%CI)** | | |
| --- | --- | --- | --- | --- | --- | --- | --- | --- | --- | --- | --- | --- | --- |
|  | **3M^c^** | ***P*** | **6M** | ***P*** | **12M** | ***P*** | **18M** | ***P*** | **24M** | ***P*** | | **3M to 24M** | ***P*** |
| **HbA_1c_^d^ (%):** F=99.9: *P*<.001; ICC^e^ PHCP^f^=0.01; ICC subject|PHCP=0.39 | | | | | | | | | | | | | |
| PTI^g^ | -0.27  (-0.45, -0.10) | .002 | -0.26  (-0.44, -0.08) | .005 | -0.17  (-0.37, 0.01) | .07 | -0.06  (-0.25, 0.13) | .52 | -0.04  (-0.23, 0.15) | .70 | | -0.15  (-0.30, 0.004) | .06 |
| PFI^h^ | -0.11  (-0.28, 0.07) | .23 | -0.17  (-0.34, 0.01) | .06 | 0.07  (-0.10, 0.25) | .42 | -0.17  (-0.35, 0.02) | .07 | 0.03  (-0.15, 0.21) | .77 | | -0.07  (-0.21, 0.08) | .38 |
| CBI^i^ | -0.10  (-0.28, 0.07) | .26 | -0.15  (-0.33, 0.03) | .09 | -0.10  (-0.28, 0.08) | .28 | -0.16  (-0.35, 0.03) | .10 | 0.05  (-0.14, 0.24) | .61 | | -0.11  (-0.26, 0.04) | .17 |
| **BMI (kg/m^2^):** F=732.9 *P*<.001; ICC PHCP = 0.01; ICC subject|PHCP=0.59 | | | | | | | | | | | | | |
| PTI | -0.34  (-0.65, -0.03) | .03 | -0.28  (-0.60, 0.04) | .08 | -0.06  (-0.39, 0.27) | .71 | -0.26  (-0.59, 0.08) | .14 | -0.15  (-0.49, 0.18) | .37 | | -0.20  (-0.48, 0.09) | .17 |
| PFI | -0.05  (-0.35, 0.24) | .72 | -0.06  (-0.35, 0.24) | .71 | 0.08  (-0.22, 0.38) | .61 | -0.29  (-0.59, 0.02) | .07 | -0.12  (-0.43, 0.19) | .45 | | -0.09  (-0.35, 0.17) | .49 |
| CBI | -0.09  (-0.40, 0.22) | .57 | 0.04  (-0.27, 0.35) | .80 | 0.27  (-0.05, 0.59) | .09 | 0.05  (-0.28, 0.38) | .76 | 0.11  (-0.22, 0.44) | .52 | | 0.11  (-0.17, 0.39) | .44 |
| **Systolic blood pressure (mm Hg):** F=60.18 *P*<.001; ICC PHCP =0.01 ICC; subject|PHCP=0.28 | | | | | | | | | | | | | |
| PTI | -2.06  (-4.66, 0.55) | .12 | 0.06  (-2.61, 2.73) | .96 | -1.96  (-4.60, 0.68) | .15 | -1.76  (-4.53, 1.02) | .21 | -2.10  (-4.80, 0.61) | .13 | | -1.49  (-3.62, 0.63) | .17 |
| PFI | -5.30  (-7.77, -2.82) | <.001 | -0.85  (-3.45, 1.66) | .51 | -1.92  (-4.41, 0.58) | .13 | -3.77  (-6.29, -1.26) | .003 | -1.87  (-4.40, 0.66) | .15 | | -2.45  (-4.46, -0.44) | .02 |
| CBI | -1.50  (-4.13, 1.14) | .27 | 0.81  (-1.88, 3.49) | .6 | -2.17  -4.88, 0.54) | .12 | -2.29  (-4.97, 0.38) | .09 | -4.43  (-7.30, -1.56) | .003 | | -1.84  (-3.95, 0.27) | .09 |
| **Diastolic blood pressure (mm Hg):** F=48.8 *P*<.001; ICC PHCP =0.02 ICC; subject|PHCP=0.25 | | | | | | | | | | | | | |
| PTI | -1.62  (-3.50, 0.25) | .09 | -1.19  (-3.10, 0.70) | .22 | -1.10  (-3.01, 0.81) | .26 | 0.40  (-1.53, 2.33) | .68 | -1.75  (-3.63, 0.13) | .07 | | -0.82  (-2.43, 0.79) | .32 |
| PFI | -2.48  (-4.29, -0.66) | .008 | -1.44  (-3.27, 0.40) | .13 | -1.65  (-3.45, 0.16) | .07 | -1.37  (-3.21, 0.47) | .14 | -2.59  (-4.41, -0.77) | .005 | | -1.72  (-3.29, -0.15) | .03 |
| CBI | -0.37  (-2.26, 1.53) | .71 | -1.80  (-3.70, 0.10) | .06 | -1.97  (-3.87, -0.07) | .04 | -1.38  (-3.28, 0.51) | .15 | -4.50  (-6.43, -2.59) | <.001 | | -2.01  (-3.63, -0.39) | .01 |
| **Waist circumference (cm):** *F=331.7 P<.001; ICC PHCP =0.02; ICC subject|PHCP=0.49* | | | | | | | | | | | | | |
| PTI | -0.56  (-1.85, 0.73) | .40 | 0.07  (-1.28, 1.42) | .92 | -0.58  (-1.92, 0.76) | .40 | -0.52  (-1.85, 0.82) | .45 | -0.69  (-2.03, 0.65) | .31 | | -0.44  (-1.62, 0.75) | .47 |
| PFI | -0.17  (-1.42, 1.08) | .79 | -2  (-3.27, -0.72) | .002 | 0.08  (-1.19, 1.35) | .90 | -0.51  (-1.79, 0.76) | .43 | -1.2  (-2.5, 0.1) | .07 | | -0.73  (-1.88, 0.41) | .21 |
| CBI | -0.6  (-1.88, 0.68) | .36 | -0.71  (-2.04, 0.63) | .30 | -0.15  (-1.49, 1.19) | .83 | -0.98  (-2.31, 0.35) | .15 | -0.56  (-1.91, 0.78) | .41 | | -0.6  (-1.79, 0.59) | .32 |
| **Weight (kg):** *F=977.2 P<.001; ICC PHCP =0.01; ICC subject|PHCP=0.59* | | | | | | | | | | | | | |
| PTI | -0.98  (-1.8, -0.14) | .02 | -0.83  (-1.7, 0.01) | .05 | -0.19  (-1.1, 0.69) | .68 | -0.67  (-1.6, 0.23) | .15 | -0.42  (-1.3, 0.48) | .36 | | -0.55  (-1.3, 0.2) | .15 |
| PFI | -0.33  (-1.1, 0.46) | .42 | -0.35  (-1.2, 0.44) | .38 | 0.03  (-0.78, 0.84) | .94 | -0.92  (-1.7, -0.1) | .03 | -0.49  (-1.3, 0.34) | .25 | | -0.42  (-1.1, 0.28) | .24 |
| CBI | -0.31  (-1.13, 0.51) | .45 | -0.002  (-0.84, 0.84) | .99 | 0.64  (-0.21, 1.5) | .14 | 0.06  (-0.83, 0.95) | 0.90 | 0.24  (-0.66, 1.14) | .61 | | 0.21  (-0.54, 0.96) | .58 |
| **Waist-to-hip ratio:** *F=92.1 P<.001; ICC PHCP =0.02; ICC subject|PHCP=0.44* | | | | | | | | | | | | | |
| PTI | 0.001  (-0.01, 0.01) | .85 | 0.004  (-0.01, 0.01) | .46 | 0.005  (-0.01, 0.01) | .35 | 0.008  (0, 0.02) | .12 | 0.005  (-0.01, 0.02) | .34 | | 0.01  (0, 0.01) | .24 |
| PFI | 0.005  (0, 0.02) | .28 | 0.006  (0, 0.02) | .22 | 0.01  (0, 0.02) | .05 | 0.005  (-0.01, 0.01) | .35 | 0.011  (0, 0.02) | .03 | | 0.01  (0, 0.02) | .09 |
| CBI | 0.004  (-0.01, 0.01) | .48 | 0.008  (0, 0.02) | .14 | 0.01  (0, 0.02) | .049 | 0.007  (0, 0.02) | .19 | 0.008  (0, 0.02) | .14 | | 0.01  (0, 0.02) | .08 |
|  |  |  | **6M** | ***P*** | **12M** | ***P*** |  |  | **24M** | ***P*** | | **6M to 24M** | ***P*** |
| **Total cholesterol (mg/dL):** F= 101.3 *P*<.001; ICC PHCP =0.01; ICC subject|PHCP=0.35 | | | | | | | | | | | | | |
| PTI |  |  | -0.40  (-6.1, 5.3) | .89 | 3.5  (-2.1, 9.0) | .22 |  |  | 2.8  (-2.1, 8.4) | .32 | | 2.6  (-2.1, 7.4) | .28 |
| PFI |  |  | -2.2  (-7.6, 3.2) | .42 | 2.1  (-3.1, 7.3) | .43 |  |  | -2.9  (-8.3, 2.6) | .30 | | -0.28  (-4.9, 4.3) | .91 |
| CBI |  |  | -2.5  (-8.1, 3.1) | .38 | 1.7  (-3.8, 7.3) | .54 |  |  | -2.9  (-8.4, 2.7) | .31 | | -0.51  (-5.2, 4.2) | .83 |
| **LDL^k^ (mg/dL):** F=108.8 *P*<.001; ICC PHCP =0.01; ICC subject|PHCP=0.34 | | | | | | | | | | | | | |
| PTI |  |  | 2.8  (-1.8, 7.5) | .24 | 3.0  (-1.6, 7.7) | .20 |  |  | 2.0  (-2.6, 6.7) | .39 | | 2.7  (-1.2, 6.5) | .18 |
| PFI |  |  | -3.5  (-7.8, 0.87) | .12 | 2.3  (-1.9, 6.6) | .28 |  |  | -2.8  (-7.3, 1.7) | .22 | | -0.34 (-4.0, 3.4) | .86 |
| CBI |  |  | -1.2  (-5.8, 3.3) | .59 | 3.7  (-0.96, 8.3) | .12 |  |  | -1.4  (-6.1, 3.3) | .56 | | 1.2 (-2.7, 5.1) | .56 |
| **HDL^l^ (mg/dL):** F=302.91 *P<*.001; ICC PHCP =0.03; ICC subject|PHCP=0.43 | | | | | | | | | | | | | |
| PTI |  |  | 1.1  (-0.56, 2.8) | .19 | 0.70  (-0.97, 2.4) | .41 |  |  | 1.2  (-0.55, 2.8) | .18 | | 0.92  (-0.62, 2.5) | .24 |
| PFI |  |  | 0.91  (-0.71, 2.5) | .27 | 0.15  (-1.5, 1.8) | .85 |  |  | 1.9  (0.28, 3.6) | .02 | | 0.87  (-0.63, 2.4) | .26 |
| CBI |  |  | 0.82  (-0.82, 2.5) | .33 | -0.80  (-2.5, 0.86) | .35 |  |  | -0.24  (-1.9, 1.5) | .78 | | -0.34  (-1.9, 1.2) | .66 |
| **Triglycerides (mg/dL):** F=46.4 *P*<.001; ICC PHCP =0.003; ICC subject|PHCP=0.41 | | | | | | | | | | | | | |
| PTI |  |  | -14.7  (-29.9, 0.46) | .05 | -2.6  (-16.9, 11.8) | .73 |  |  | -7.3  (-22.2, 7.5) | .33 | | -6.2  (-18.2, 5.8) | .31 |
| PFI |  |  | 7.2  (-7.8, 22.2) | .35 | -1.1  (-14.6, 12.3) | .87 |  |  | -10.9  (-24.8, 2.9) | .12 | | -3.0  (-14.4, 8.4) | .61 |
| CBI |  |  | -1.6  (-16.2, 12.9) | .83 | -2.0  (-16.0, 12.1) | .79 |  |  | 3.3  (-11.5, 18.2) | .66 | | -0.14  (-12.0, 11.7) | .98 |
| **Fasting serum glucose** **(mg/dL):** F=87.1 *P*<.001; ICC PHCP =0.01; ICC subject|PHCP=0.31 | | | | | | | | | | | | | |
| PTI |  |  | -5.6  (-12.2, 1.0) | .099 | -1.6  (-8.2, 5.0) | .63 |  |  | 0.83  (-6.1, 7.8) | .82 | | -1.5  (-7.0, 4.0) | .60 |
| PFI |  |  | -9.4  (-15.7, -3.1) | .004 | -1.9  (-8.1, 4.3) | .56 |  |  | 3.5  (-3.0, 10.0) | .29 | | -1.3  (-6.6, 3.9) | .62 |
| CBI |  |  | -6.3  (-12.7, 0.2) | .05 | -4.9  (-11.4, 1.6) | .14 |  |  | 4.1  (-2.9, 11.1) | .25 | | -2.1  (-7.6, 3.3) | .44 |
|  |  |  |  |  | **12M** | ***P*** |  |  | **24M** | ***P*** | | **12M to 24M** | ***P*** |
| **Serum** **Creatinine (mg/dL):** F=407.1 *P*<.001; ICC PHCP =0.21; ICC subject|PHCP=0.53 | | | | | | | | | | | | | |
| PTI |  |  |  |  | -0.01  (-0.07, 0.05) | .74 |  |  | -0.04  (-0.1, 0.03) | .25 | | -0.02  (-0.09, 0.04) | .45 |
| PFI |  |  |  |  | -0.02  (-0.09, 0.04) | .46 |  |  | -0.04  (-0.10, 0.02) | .21 | | -0.03  (-0.09, 0.03) | .32 |
| CBI |  |  |  |  | 0.02  (-0.04, 0.09) | .44 |  |  | 0.002  (-0.06, 0.06) | .96 | | 0.01  (-0.05, 0.08) | .68 |
| **Glomerular filtration rate (mL/min)**: F=297.6 *P*<.001; ICC PHCP =0.24; ICC subject|PHCP=0.56 | | | | | | | | | | | | | |
| PTI |  |  |  |  | 1.2  (-6.9, 9.3) | .78 |  |  | 4.7  (-3.4, 12.8) | .26 | | 2.9  (-5.1, 11) | .47 |
| PFI |  |  |  |  | 2.4  (-5.7, 10.4) | .57 |  |  | 5.5  (-2.6, 13.6) | .19 | | 3.9  (-4.1, 11.9) | .34 |
| CBI |  |  |  |  | -4.0  (-12.1, 4.1) | .34 |  |  | 0.54  (-7.6, 8.7) | .90 | | -1.7  (-9.8, 6.3) | .68 |
| ^a^UC: usual care or control group.  ^b^AUC: area under the curve.  ^c^M: months.  ^d^HbA_1c_: glycated hemoglobin  ^e^ICC: Intraclass correlation coefficient.  ^f^PHCP: Primary Care Health Practices.  ^g^PTI is an intervention only for patients and family members.  ^h^PFI is an intervention only for health care professionals at primary care.  ^i^CBI is a combined intervention for patients and professionals.  ^j^LDL: low-density lipoprotein.  ^k^HDL: high-density lipoprotein. | | | | | | | | | | | | | |
