# Supplementary material for: Effectiveness of Internet-Based Multicomponent Interventions for Patients and Health Care Professionals to Improve Clinical Outcomes in Type 2 Diabetes Evaluated Through the INDICA Study: Multiarm Cluster Randomized Controlled Trial
Source: JMIR Mhealth Uhealth. 2020 Nov 2;8(11):e18922. doi: 10.2196/18922 (PMC7669446; doi:10.2196/18922)
Supplement: Multimedia Appendix 5 [file mhealth_v8i11e18922_app5.doc]

Multimedia Appendix 5. Adjusted means for each group and intragroup differences compared with the baseline measurement for the whole sample

|  | **Adjusted means in each group (95%CI)** | | | | | | | | | | | | | | | | | | | **Difference in intragroup of adjusted means compared to baseline (95%CI)** | | | | | | | | | | | | | |
| --- | --- | --- | --- | --- | --- | --- | --- | --- | --- | --- | --- | --- | --- | --- | --- | --- | --- | --- | --- | --- | --- | --- | --- | --- | --- | --- | --- | --- | --- | --- | --- | --- | --- |
|  | **B^d^** | | | **3M^e^** | | **6M** | | | **12M** | | | **18M** | | | **24M** | | | **3M-B** | | | **6M-B** | | | **12M-B** | | | | **18M-B** | | | **24M-B** | | |
| **HbA_1c_^f^ (%)** | | | | | | | | | | | | | | | | | | | | | | | | | | | | | | | | | |
| PTI^g^ | | | 7.3  (7.3, 7.4) | 7.0  (6.8, 7.1) | | 7.1  (7.0, 7.2) | | | 7.1  (7.0, 7.3) | | | 7.4  (7.2, 7.5) | | | 7.3  (7.2, 7.4) | | | -0.35  (-0.5, -0.2)^a^ | | | -0.24  (-0.4, -0.1)^b^ | | | -0.20  (-0.3, -0.07)^b^ | | | | 0.05  (-0.09, 0.2) | | | -0.03  (-0.2, 0.1) | | |
| PFI^h^ | | | 7.2  (7.1, 7.3) | 7.1  (7.0, 7.3) | | 7.2  (7.1, 7.3) | | | 7.4  (7.2, 7.5) | | | 7.3  (7.1, 7.4) | | | 7.4  (7.2, 7.5) | | | -0.02  (-0.1, -0.1)^b^ | | | 0.02  (-0.1, 0.1) | | | 0.21  (0.08, 0.3)^b^ | | | | 0.11  (-0.02, 0.2)^c^ | | | 0.20  (0.07, 0.3)^b^ | | |
| CBI^i^ | | | 7.4  (7.3, 7.4) | 7.1  (7.0, 7.3) | | 7.2  (7.1, 7.3) | | | 7.2  (7.1, 7.3) | | | 7.3  (7.1, 7.4) | | | 7.4  (7.2, 7.5) | | | -0.22  (-0.4, -0.09)^b^ | | | -0.17  (-0.3, -0.04)^b^ | | | -0.17  (-0.3, -0.04)^b^ | | | | -0.09  (-0.2, 0.05) | | | 0.01  (-0.1, 0.2) | | |
| UC^j^ | | | 7.3  (7.2, 7.3) | 7.2  (7.1, 7.4) | | 7.4  (7.2, 7.5) | | | 7.3  (7.2, 7.4) | | | 7.4  (7.3, 7.6) | | | 7.3  (7.2, 7.5) | | | -0.02  (-0.1, 0.1) | | | 0.08  (-0.04, 0.2) | | | 0.03  (-0.09, 0.2) | | | | 0.17  (0.04, 0.3)^b^ | | | 0.07  (-0.06, 0.2) | | |
| **BMI (kg/m^2^)** | | | | | | | | | | | | | | | | | | | | | | | | | | | | | | | | | |
| PTI | | | 31.6  (31.4, 31.8) | 31.7  (31.5, 31.9) | | 31.8  (31.5, 32.0) | | | 31.7  (31.4, 31.9) | | | 31.7  (31.5, 32.0) | | | 31.6  (31.3, 31.9) | | | -0.09  (-0.1, 0.3) | | | 0.14  (-0.1, 0.4) | | | 0.06  (-0.2, 0.3) | | | | 0.10  (-0.2, 0.4) | | | -0.02  (-0.3, 0.6) | | |
| PFI | | | 32.4  (32.2, 32.6) | 32.0  (31.8, 32.2) | | 32.0  (31.8, 32.2) | | | 31.8  (31.6, 32.0) | | | 31.7  (31.5, 31.9) | | | 31.6  (31.4, 31.9) | | | -0.42  (-0.6, -0.2)^a^ | | | -0.43  (-0.6, -0.2)^a^ | | | -0.59  (-0.8, -0.4)^a^ | | | | -0.73  (-0.9, -0.5)^a^ | | | -0.78  (-1.0, -0.6)^a^ | | |
| CBI | | | 32.1  (31.9, 32.3) | 32.0  (31.7, 32.2) | | 32.1  (31.9, 32.3) | | | 32.0  (31.8, 32.3) | | | 32.0  (31.8, 32.3) | | | 31.9  (31.6, 32.1) | | | -0.11  (-0.3, 0.1) | | | 0.02  (-0.2, 0.3) | | | -0.05  (-0.3, 0.2) | | | | -0.04  (-0.3, 0.2) | | | -0.20  (-0.4, 0.05) | | |
| UC | | | 32.1  (31.9, 32.3) | 32.1  (31.8, 32.3) | | 32.1  (31.8, 32.3) | | | 31.8  (31.5, 32.0) | | | 32.0  (31.8, 32.2) | | | 31.8  (31.5, 32.0) | | | -0.02  (-0.2, 0.2) | | | -0.03  (-0.2, 0.2) | | | -0.32  (-0.5, -0.1)^b^ | | | | -0.09  (-0.3, 0.1) | | | -0.31  (-0.5, -0.09)^b^ | | |
| **Systolic blood pressure (mm Hg)** | | | | | | | | | | | | | | | | | | | | | | | | | | | | | | | | | |
| PTI | | | 132.8  (132.1, 133.6) | | 128.4  (126.5, 130.3) | | 131.4  (129.5, 133.3) | | | 129.2  (127.3, 131.1) | | | 130.8  (128.7, 132.8) | | | 127.6  (125.7, 129.6) | | | -4.4  (-6.3, -2.5)^a^ | | | -1.5  (-3.4, 0.5) | | | -3.7  (-5.6, -1.8)^a^ | | | | -2.1  (-4.1, -0.03)^b^ | | | -5.2  (-7.2, -3.2)^a^ | |
| PFI | | | 133.4  (132.7, 134.0) | | 125.2  (123.5, 126.9) | | 130.5  (128.8, 132.2) | | | 129.2  (127.5, 130.9) | | | 128.8  (127.0, 130.5) | | | 127.9  (126.1, 129.7) | | | -8.2  (-9.9, -6.5)^a^ | | | -2.9  (-4.6, -1.2)^b^ | | | -4.2  (-5.9, -2.4)^a^ | | | | -4.6  (-6.3, -2.9)^a^ | | | -5.5  (-7.3, -3.7)^a^ | |
| CBI | | | 132.8  (131.9, 133.6) | | 129.0  (127.1, 130.9) | | 132.1  (130.3, 134.0) | | | 129.0  (127.0, 130.9) | | | 130.2  (128.3, 132.2) | | | 125.3  (123.0, 127.6) | | | -3.8  (-5.7, -1.9)^a^ | | | -0.64  (-2.5, 1.2) | | | -3.8  (-5.8, -1.9)^a^ | | | | -2.5  (-4.5, -0.6)^b^ | | | -7.5  (-9.8, -5.2)^a^ | |
| UC | | | 132.6  (131.9, 133.3) | | 130.5  (128.7, 132.3) | | 131.3  (129.5, 133.2) | | | 131.1  (129.3, 132.9) | | | 132.5  (130.7, 134.4) | | | 129.7  (127.9, 131.5) | | | -2.1  (-3.9, -.3)^b^ | | | -1.3  (-3.1, 0.6) | | | -1.5  (-3.3, 0.3) | | | | -0.08  (-1.9, 1.8) | | | -2.9  (-4.7, -1.1)^b^ | |
| **Diastolic blood pressure (mm Hg)** | | | | | | | | | | | | | | | | | | | | | | | | | | | | | | | | | |
| PTI | | | 84.2  (83.7, 84.6) | 80.8  (79.4, 82.1) | | 83.0  (81.6, 84.3) | | | 82.2  (80.8, 83.6) | | | 83.2  (81.8, 84.6) | | | 80.7  (79.4, 82.1) | | | -3.4  (-4.7, -2.0)^a^ | | | -1.2  (-2.5, 0.2)^c^ | | | -2.0  (-3.3, -0.6)^b^ | | | | -0.95  (-2.3, 0.4) | | | -3.4  (-4.8, -2.1)^a^ | | |
| PFI | | | 84.5  (84.1, 84.9) | 79.9  (78.6, 81.2) | | 82.7  (81.5, 84.0) | | | 81.7  (80.4, 82.9) | | | 81.4  (80.1, 82.7) | | | 79.9  (78.6, 81.2) | | | -4.6  (-5.9, -3.3)^a^ | | | -1.8  (-3.1, -0.5)^b^ | | | -2.8  (-4.1, -1.6)^a^ | | | | -3.1  (-4.4, -1.8)^a^ | | | -4.6  (-5.9, -3.3)^a^ | | |
| CBI | | | 84.7  (84.2, 85.2) | 82.0  (80.6, 83.4) | | 82.4  (81.0, 83.7) | | | 81.3  (80.0, 82.7) | | | 81.4  (80.0, 82.8) | | | 78.0  (76.5, 79.4) | | | -2.7  (-4.1, -1.3)^a^ | | | -2.3  (-3.7, -1.0)^b^ | | | -3.4  (-4.7, -2.0)^a^ | | | | -3.3  (-4.7, -1.9)^a^ | | | -6.7  (-8.2, -5.3)^a^ | | |
| UC | | | 83.8  (83.4, 84.1) | 82.4  (81.1, 83.7) | | 84.2  (82.8, 85.5) | | | 83.3  (82.0, 84.6) | | | 82.8  (81.5, 84.1) | | | 82.5  (81.2, 83.8) | | | -1.4  (-2.7, -0.08)^b^ | | | -0.40  (-0.9, 1.7) | | | -0.47  (-1.8, 0.8) | | | | -1.0  (-2.3, 0.3) | | | -1.3  (-2.6, -0.004)^b^ | | |
| **Waist circumference (cm)** | | | | | | | | | | | | | | | | | | | | | | | | | | | | | | | | | |
| PTI | | | 104.8  (104.3, 105,3) | 105.4  (104.4, 106.3) | | 106.1  (105.1, 107.1) | | | 105  (104, 106) | | | 105.4  (104.5, 106.4) | | | 105.1  (104.2, 106.1) | | | 0.58  (-0.34, 1.51) | | | 1.33  (0.35, 2.3)^b^ | | | 0.21  (-0.75, 1.2) | | | | 0.66  (-0.32, 1.6) | | | 0.37  (-0.61, 1.4) | | |
| PFI | | | 106.7  (106.2, 107.1) | 105.7  (104.9, 106.6) | | 104  (103.1, 104.9) | | | 105.6  (104.7, 106.5) | | | 105.4  (104.5, 106.3) | | | 104.6  (103.7, 105.5) | | | -0.91  (-1.8, -0.04)^b^ | | | -2.63  (-3.5, -1.7)^a^ | | | -1.01  (-1.9, -0.11)^b^ | | | | -1.23  (-2.2, -0.33)^b^ | | | -2.02  (-2.9, -1.1)^a^ | | |
| CBI | | | 105.4  (104.9, 105.9) | 105.3  (104.4, 106.2) | | 105.3  (104.4, 106.3) | | | 105.4  (104.4, 106.4) | | | 105  (104, 105.9) | | | 105.3  (104.3, 106.2) | | | -0.12  (-1.04, 0.8) | | | -0.12  (-1.08, 0.85) | | | -0.02  (-1, 0.95) | | | | -0.48  (-1.4, 0.48) | | | -0.17  (-1.1, 0.81) | | |
| UC | | | 105.8  (105.3, 106.2) | 105.9  (105, 106.8) | | 106  (105.1, 106.9) | | | 105.6  (104.6, 106.5) | | | 105.9  (105, 106.9) | | | 105.8  (104.9, 106.8) | | | 0.13  (-0.76, 1.03) | | | 0.25  (-0.66, 1.2) | | | -0.22  (-1.1, 0.7) | | | | 0.16  (-0.75, 1.1) | | | 0.05  (-0.87, 0.98) | | |
| **Weight (kg)** | | | | | | | | | | | | | | | | | | | | | | | | | | | | | | | | | |
| PTI | | | 86  (84.5, 87.5) | 85.8  (85.2, 86.4) | | 85.9  (85.3, 86.6) | | | 85.8  (85.1, 86.4) | | | 85.9  (85.2, 86.6) | | | 85.5  (84.8, 86.2) | | | -0.94  (-1.6, -0.33)^b^ | | | -0.78  (-1.4, -0.16)^b^ | | | -0.95  (-1.6, -0.29)^b^ | | | | -0.84  (-1.5, -0.14)^b^ | | | -1.2  (-1.9, -0.46)^b^ | | |
| PFI | | | 87  (85.7, 88.4) | 86.4  (85.9, 87) | | 86.4  (85.8, 87) | | | 86  (85.4, 86.5) | | | 85.6  (85, 86.2) | | | 85.5  (84.9, 86.1) | | | -0.29  (-0.84, 0.26) | | | -0.3  (-0.86, 0.25) | | | -0.74  (-1.3, -0.17)^b^ | | | | -1.1  (-1.7, -0.52)^a^ | | | -1.2  (-1.8, -0.65)^a^ | | |
| CBI | | | 86.8  (85.4, 88.2) | 86.4  (85.8, 87) | | 86.8  (86.1, 87.4) | | | 86.6  (86, 87.2) | | | 86.6  (85.9, 87.3) | | | 86.2  (85.5, 86.9) | | | -0.27  (-0.86, 0.31) | | | 0.05  (-0.56, 0.66) | | | -0.13  (-0.75, 0.5) | | | | -0.11  (-0.77, 0.54) | | | -0.51  (-1.2, 0.16) | | |
| UC | | | 86.9  (85.5, 88.4) | 86.7  (86.2, 87.3) | | 86.8  (86.2, 87.3) | | | 85.9  (85.4, 86.5) | | | 86.5  (85.9, 87.1) | | | 86.0  (85.4, 86.6) | | | 0.04  (-0.53, 0.61) | | | 0.05  (-0.52, 0.62) | | | -0.76  (-1.35, -0.18)^b^ | | | | -0.17  (-0.76, 0.43) | | | -0.74  (-1.34, -0.15)^b^ | | |
| **Waist-to-hip ratio** | | | | | | | | | | | | | | | | | | | | | | | | | | | | | | | | | |
| PTI | | | 0.99  (0.98, 0.99) | 0.98  (0.97, 0.98) | | 0.98  (0.97, 0.99) | | | 0.98  (0.97, 0.99) | | | 0.99  (0.98, 1) | | | 0.99  (0.98, 0.99) | | | -0.01  (-0.02, -0.001)^b^ | | | -0.01  (-0.01, 0.002) | | | -0.003  (-0.01, 0.004) | | | | 0.005  (0, 0.01) | | | 0.001  (-0.01, 0.01) | | |
| PFI | | | 0.97  (0.97, 0.98) | 0.98  (0.98, 0.99) | | 0.98  (0.98, 0.99) | | | 0.99  (0.98, 0.99) | | | 0.99  (0.98, 0.99) | | | 0.99  (0.99, 1) | | | 0.01  (0.001, 0.01)^b^ | | | 0.01  (0.002, 0.02)^b^ | | | 0.01  (0.01, 0.02)^a^ | | | | 0.01  (0.01, 0.02)^a^ | | | 0.02  (0.01, 0.03)^a^ | | |
| CBI | | | 0.98  (0.98, 0.98) | 0.98  (0.97, 0.99) | | 0.98  (0.98, 0.99) | | | 0.99  (0.98, 1) | | | 0.99  (0.98, 1) | | | 0.99  (0.98, 1) | | | 0.001  (-0.01, 0.01) | | | 0.005  (0, 0.01) | | | 0.01  (0, 0.02)^b^ | | | | 0.01  (0, 0.02)^b^ | | | 0.01  (0, 0.02)^b^ | | |
| UC | | | 0.99  (0.99, 0.99) | 0.98  (0.97, 0.98) | | 0.98  (0.97, 0.98) | | | 0.98  (0.97, 0.98) | | | 0.98  (0.98, 0.99) | | | 0.98  (0.97, 0.99) | | | -0.01  (-0.02, -0.01)^a^ | | | -0.01  (-0.02, -0.01)^a^ | | | -0.01  (-0.02, -0.01)^a^ | | | | -0.01  (-0.02, -0.002)^b^ | | | -0.01  (-0.02, -0.003)^b^ | | |
|  | **B** | | |  | | **6M** | | | **12M** | | |  | | | **24M** | | |  | | | **6M-B** | | | **12M-B** | | | |  | | | **24M-B** | | |
| **Total cholesterol (mg/dL)** | | | | | | | | | | | | | | | | | | | | | | | | | | | | | | | | | |
| PTI | | | 189.6  (188.1, 191.1) |  | | 186.5  (182.3, 190.7) | | | 187.1  (183.1, 191.2) | | |  | | | 185.2  (181.1, 189.3) | | |  | | | -3.1  (-7.3, 1) | | | -2.5  (-6.5, 1.6) | | | |  | | | -4.4  (-8.6, -0.28)^b^ | | |
| PFI | | | 188.7  (187.3, 190.1) |  | | 184.7  (181, 188.4) | | | 185.7  (182.1, 189.4) | | |  | | | 179.5  (175.7, 183.3) | | |  | | | -4  (-7.7, -0.30)^b^ | | | -3  (-6.6, 0.66) | | | |  | | | -9.2  (-13, -5.4)^a^ | | |
| CBI | | | 189.5  (187.9, 191.1) |  | | 184.4  (180.4, 188.3) | | | 185.4  (181.3, 189.4) | | |  | | | 179.5  (175.4, 183.5) | | |  | | | -5.1  (-9.1, -1.2)^b^ | | | -4.1  (-8.1, -0.07)^b^ | | | |  | | | -10  (-14.1, -6.0)^a^ | | |
| UC | | | 186.6  (185.2, 188.1) |  | | 186.9  (182.9, 190.8) | | | 183.6  (179.9, 187.4) | | |  | | | 182.4  (178.5, 186.2) | | |  | | | 0.3  (-3.7, 4.2) | | | -3  (-6.7, 0.77) | | | |  | | | -4.3  (-8.2, -0.39)^b^ | | |
| **LDL^k^ (mg/dL)** | | | | | | | | | | | | | | | | | | | | | | | | | | | | | | | | | |
| PTI | | | 109.1  (107.8, 110.5) |  | | 110.2  (106.8, 113.6) | | | 106.3  (103, 109.7) | | |  | | | 105.8  (102.4, 109.2) | | |  | | | 1  (-2.4, 4.5) | | | -2.8  (-6.2, 0.5) | | | |  | | | -3.4  (-6.8, 0)^c^ | | |
| PFI | | | 108.9  (107.7, 110.2) |  | | 103.9  (100.9, 107) | | | 105.7  (102.7, 108.6) | | |  | | | 100.9  (97.8, 104) | | |  | | | -5  (-8.1, -2)^b^ | | | -3.3  (-6.3, -0.3)^b^ | | | |  | | | -8  (-11.1, -4.9)^a^ | | |
| CBI | | | 107.8  (106.4, 109.3) |  | | 106.1  (102.9, 109.4) | | | 107  (103.6, 110.4) | | |  | | | 102.3  (98.9, 105.8) | | |  | | | -1.7  (-5, 1.6) | | | -0.9  (-4.2, 2.5) | | | |  | | | -5.5  (-9, -2.1)^b^ | | |
| UC | | | 108.4  (107.1, 109.8) |  | | 107.4  (104.2, 110.5) | | | 103.3  (100.2, 106.4) | | |  | | | 103.7  (100.5, 106.9) | | |  | | | -1.1  (-4.2, 2.1) | | | -5.1  (-8.2, -2)^b^ | | | |  | | | -4.7  (-7.9, -1.5)^b^ | | |
| **HDL^l^ (mg/dL)** | | | | | | | | | | | | | | | | | | | | | | | | | | | | | | | | | |
| PTI | | 49.2  (48.7, 49.6) | |  | | 49.2  (48, 50.5) | | | 49.1  (47.9, 50.4) | | |  | | | 48.3  (47.1, 49.6) | | |  | | | 0.05  (-1.19, 1.3) | | | -0.03  (-1.3, 1.2) | | | |  | | | -0.82  (-2.0, 0.41) | | |
| PFI | | 48.6  (48.1, 49) | |  | | 49  (47.8, 50.1) | | | 48.6  (47.5, 49.7) | | |  | | | 49.1  (48, 50.3) | | |  | | | 0.40  (-0.73, 1.5) | | | 0.01  (-1.1, 1.1) | | | |  | | | 0.54  (-0.62, 1.7) | | |
| CBI | | 49.2  (48.7, 49.7) | |  | | 48.9  (47.7, 50.1) | | | 47.6  (46.4, 48.9) | | |  | | | 47  (45.7, 48.2) | | |  | | | -0.30  (-1.5, 0.89) | | | -1.6  (-2.8, -0.34)^b^ | | | |  | | | -2.2  (-3.5, -1.0)^a^ | | |
| UC | | 47.6  (47.1, 48.1) | |  | | 48.1  (46.9, 49.2) | | | 48.4  (47.3, 49.6) | | |  | | | 47.2  (46, 48.4) | | |  | | | 0.46  (-0.69, 1.6) | | | 0.82  (-0.32, 2.0) | | | |  | | | -0.42  (-1.6, 0.75) | | |
| **Triglycerides (mg/dL)** | | | | | | | | | | | | | | | | | | | | | | | | | | | | | | | | | |
| PTI | | 166.9  (162.2, 171.7) | |  | | 150.6  (139.8, 161.4) | | | 164.9  (153.2, 174.7) | | |  | | | 157.4  (146.6, 168.2) | | |  | | | -16.3  (-27.1, -5.5)^b^ | | | -3  (-13.8, 7.8) | | | |  | | | -9.6  (-20.4, 1.2)^c^ | | |
| PFI | | 160.1  (156.8, 163.3) | |  | | 172.5  (162.1, 183) | | | 165.4  (156.1, 174.7) | | |  | | | 153.8  (144.2, 163.3) | | |  | | | 12.5  (2.1, 22.9)^b^ | | | 5.4  (-4, 14.7) | | | |  | | | -6.3  (-15.8, 3.2) | | |
| CBI | | 165.5  (161.4, 169.6) | |  | | 163.7  (153.5, 173.8) | | | 164.6  (154.3, 174.8) | | |  | | | 168  (157.3, 178.7) | | |  | | | -1.8  (-12, 8.3) | | | -0.9  (-11.2, 9.3) | | | |  | | | 2.5  (-8.2, 13.2) | | |
| UC | | 158.4  (154.5, 162.3) | |  | | 165.3  (154.7, 176) | | | 166.5  (156.8, 176.2) | | |  | | | 164.7  (154.6, 174.8) | | |  | | | 6.9  (-3.7, 17.6) | | | 8.1  (-1.6, 17.8) | | | |  | | | 6.3  (-3.8, 16.4) | | |
| **Fasting serum glucose (mg/dL)** | | | | | | | | | | | | | | | | | | | | | | | | | | | | | | | | | |
| PTI | | 153.7  (151.8, 155.7) | |  | | 150.8  (146, 155.6) | | | 151.8  (146.9, 156.7) | | |  | | | 151.5  (146.4, 156.5) | | |  | | | -2.9  (-7.8, 1.8) | | | -1.9  (-6.8, 3.0) | | | |  | | | -2.3  (-7.3, 2.7) | | |
| PFI | | 148.6  (147, 150.3) | |  | | 147  (142.6, 151.3) | | | 151.6  (147.3, 155.9) | | |  | | | 154.1  (149.6, 158.6) | | |  | | | -1.7  (-6.0, 2.7) | | | 2.9  (-1.3, 7.3) | | | |  | | | 5.5  (1.0, 10.0)^b^ | | |
| CBI | | 154  (152, 156.1) | |  | | 150.1  (145.4, 154.8) | | | 148.5  (143.8, 153.2) | | |  | | | 154.7  (149.6, 159.8) | | |  | | | -3.9  (-8.6, 0.75) | | | -5.5  (-10.2, -0.78)^b^ | | | |  | | | 0.7  (-4.4, 5.8) | | |
| UC | | 152.1  (150.3, 154) | |  | | 156.4  (151.8, 160.9) | | | 153.5  (149, 157.9) | | |  | | | 150.6  (145.9, 155.3) | | |  | | | 4.2  (-0.31, 8.8)^c^ | | | 1.3  (-3.1, 5.8) | | | |  | | | -1.5  (-6.2, 3.2) | | |
|  | **B** | | |  | |  | | | **12M** | | |  | | | **24M** | | |  | | |  | | | **12M-B** | | | |  | | | **24M-B** | | |
| **Serum Creatinine (mg/dL)** | | | | | | | | | | | | | | | | | | | | | | | | | | | | | | | | | |
| PTI | | 0.82  (0.81, 0.83) | |  | |  | | | 0.78  (0.74, 0.83) | | |  | | | 0.78  (0.73, 0.82) | | |  | | |  | | | -0.04  (-0.08, 0.01) | | | |  | | | -0.04  (-0.08, 0.00)^c^ | | |
| PFI | | 0.81  (0.8, 0.82) | |  | |  | | | 0.77  (0.73, 0.81) | | |  | | | 0.78  (0.73, 0.82) | | |  | | |  | | | -0.04  (-0.08, 0)^c^ | | | |  | | | -0.03  (-0.08, 0.01) | | |
| CBI | | 0.78  (0.77, 0.79) | |  | |  | | | 0.82  (0.77, 0.86) | | |  | | | 0.82  (0.77, 0.86) | | |  | | |  | | | 0.04  (-0.01, 0.08)^c^ | | | |  | | | 0.04  (-0.01, 0.08) | | |
| UC | | 0.78  (0.77, 0.79) | |  | |  | | | 0.79  (0.75, 0.84) | | |  | | | 0.82  (0.77, 0.86) | | |  | | |  | | | 0.01  (-0.03, 0.06) | | | |  | | | 0.03  (-0.01, 0.08) | | |
| **Glomerular filtration rate (mL/min)** | | | | | | | | | | | | | | | | | | | | | | | | | | | | | | | | | |
| PTI | | 90.0  (89.2, 90.9) | |  | |  | | | 93.4  (87.7, 99.2) | | |  | | | 92.9  (87.2, 98.7) | | |  | | |  | | | 3.4  (-2.4, 9.1) | | |  | | | | 2.9  (-2.8, 8.7) | | |
| PFI | | 88.6  (87.8, 89.3) | |  | |  | | | 94.6  (88.9,100.3) | | |  | | | 93.8  (88.0, 99.5) | | |  | | |  | | | 6.1  (0.36, 11.8)^b^ | | |  | | | | 5.2  (-0.52, 10.9)^c^ | | |
| CBI | | 93.5  (92.7, 94.4) | |  | |  | | | 88.3  (82.5, 94.0) | | |  | | | 88.8  (83.0, 94.6) | | |  | | |  | | | -5.2  (-10.9, 0.54)^c^ | | |  | | | | -4.7  (-10.5, 1.1) | | |
| UC | | 93.9  (93.1, 94.7) | |  | |  | | | 92.3  (86.5, 97.9) | | |  | | | 88.3  (82.5, 94.0) | | |  | | |  | | | -1.6  (-7.4, 4.1) | | |  | | | | -5.6  (-11.4, 0.11)^c^ | | |
| ^a^: *P*<.001.  ^b^: *P*<.05.  ^c^: *P*<.1.  ^d^B: baseline.  ^e^M: months.  ^f^HbA_1c_: glycated hemoglobin.  ^g^PTI is an intervention only for patients and family members.  ^h^PFI is an intervention only for health care professionals at primary care.  ^i^CBI is a combined intervention for patients and professionals.  ^j^UC: usual care or control group.  ^k^LDL: low-density lipoprotein.  ^l^HDL: high-density lipoprotein. | | | | | | | | | | | | | | | | | | | | | | | | | | | | | | | | | |
|  | | | | | | | |  | | |  | | |  | | |  | | |  | | |  | | |  | | | |  | | |  |
