# Supplementary material for: Effectiveness of Internet-Based Multicomponent Interventions for Patients and Health Care Professionals to Improve Clinical Outcomes in Type 2 Diabetes Evaluated Through the INDICA Study: Multiarm Cluster Randomized Controlled Trial
Source: JMIR Mhealth Uhealth. 2020 Nov 2;8(11):e18922. doi: 10.2196/18922 (PMC7669446; doi:10.2196/18922)
Supplement: Multimedia Appendix 6 [file mhealth_v8i11e18922_app6.doc]

Multimedia Appendix 6. Patients with clinically relevant changes in HbA_1c_ and comparison with the usual care group. HbA_1c_: glycated hemoglobin.

| **All the sample** | | | | | | | | | | |
| --- | --- | --- | --- | --- | --- | --- | --- | --- | --- | --- |
|  | 3M^a^ | *P* | 6M | *P* | 12M | *P* | 18M | *P* | 24M | *P* |
| PTI^b^ | 38.6% | <.001 | 33.1% | .005 | 35.6% | .006 | 29.2% | .10 | 30.2% | .21 |
| PFI^c^ | 24.4% | .20 | 26.7% | .16 | 22.8% | .27 | 27.5% | .24 | 26.1% | .99 |
| CBI^d^ | 27.8% | .03 | 30.5% | .02 | 31.6% | .09 | 32.6% | .009 | 30.8% | .14 |
| UC^e^ | 20.3% |  | 21.4% |  | 26.0% |  | 23.9% |  | 26.2% |  |
| **HbA_1c_^f^ baseline > 7%** | | | | | | | | | | |
|  | 3M | *P* | 6M | *P* | 12M | *P* | 18M | *P* | 24M | *P* |
| PTI | 57.3% | <.001 | 51.8% | .01 | 53.1% | .049 | 46.0% | .41 | 48.7% | .46 |
| PFI | 42.2% | .02 | 46.1% | .08 | 40.5% | .57 | 46.3% | .35 | 47.7% | .59 |
| CBI | 42.6% | .02 | 45.6% | .10 | 47.2% | .41 | 48.8% | .16 | 48.0% | .54 |
| UC | 31.7% |  | 36.4% |  | 43.2% |  | 41.8% |  | 45.1% |  |
| ^a^M: months.  ^b^PTI is an intervention only for patients and family members.  ^c^PFI is an intervention only for health care professionals at primary care.  ^d^CBI is a combined intervention for patients and professionals.  ^e^UC: usual care or control group.  ^f^HbA_1c_: glycated hemoglobin.  Clinically relevant changes are differences in HbA_1c_ >= 0.4%. | | | | | | | | | | |
