# Supplementary material for: Effectiveness of Internet-Based Multicomponent Interventions for Patients and Health Care Professionals to Improve Clinical Outcomes in Type 2 Diabetes Evaluated Through the INDICA Study: Multiarm Cluster Randomized Controlled Trial
Source: JMIR Mhealth Uhealth. 2020 Nov 2;8(11):e18922. doi: 10.2196/18922 (PMC7669446; doi:10.2196/18922)
Supplement: Multimedia Appendix 7 [file mhealth_v8i11e18922_app7.doc]

Multimedia Appendix 7. Adjusted difference in means and area under the curve of each group compared with the usual care group for patients with a baseline HbA_1c_ >7. HbA_1c_: glycated hemoglobin.

|  | | **Adjusted difference in intragroup means compared to the UC^a^ group: Mean (95%CI)** | | | | | | | | | | | | | | | | | | | **Difference in AUC^b^ compared to the UC group (95%CI)** | | | | | |
| --- | --- | --- | --- | --- | --- | --- | --- | --- | --- | --- | --- | --- | --- | --- | --- | --- | --- | --- | --- | --- | --- | --- | --- | --- | --- | --- |
|  | | **3M^c^** | ***P*** | | **6M** | | ***P*** | | **12M** | | | ***P*** | | **18M** | ***P*** | | **24M** | | | ***P*** | | | **3M to 24M** | ***P*** | |  |
| **HbA_1c_^d^ (%):** F=17.0: *P*<.001; ICC^e^ PHCP^f^ = 0.01; ICC subject|PHCP=0.37 | | | | | | | | | | | | | | | | | | | | | | | | | | |
| PTI^g^ | -0.53  (-0.80, -0.25) | | | <.001 | | -0.49  (-0.77, -0.20) | | .001 | | -0.29  (-0.59, 0.01) | .05 | | -0.06  (-0.36, 0.24) | | | .68 | | -0.10  (-0.40, 0.19) | .49 | | | -0.26  (-0.48, -0.04) | | | .03 | |
| PFI^h^ | -0.30  (-0.57, -0.02) | | | .03 | | -0.36  (-0.63, -0.08) | | .01 | | -0.05  (-0.32, 0.23) | .74 | | -0.24  (-0.53, 0.04) | | | .09 | | -0.07  (-0.36, 0.22) | .63 | | | -0.19  (-0.41, 0.03) | | | .09 | |
| CBI^i^ | -0.29  (-0.56, -0.02) | | | .04 | | -0.33  (-0.60, -0.05) | | .02 | | -0.26  (-0.55, 0.02) | .07 | | -0.27  (-0.56, 0.02) | | | .07 | | -0.04  (-0.34, 0.25) | .79 | | | -0.25  (-0.47, -0.03) | | | .03 | |
| **BMI (kg/m2):** F=342.2 *P*<.001; ICC PHCP = 0.01; ICC subject|PHCP=0.59 | | | | | | | | | | | | | | | | | | | | | | | | | |  |
| PTI | | -0.24  (-0.62, 0.15) | .23 | | -0.18  (-0.57, 0.20) | | .35 | | -0.01  (-0.42, 0.40) | | | .96 | | -0.4  (-0.83, 0.02) | .06 | | -0.21  (-0.64, 0.22) | | | .35 | | | -0.2  (-0.54, 0.13) | .23 | |  |
| PFI | | -0.05  (-0.42, 0.32) | .78 | | -0.02  (-0.39, 0.35) | | .91 | | -0.01  (-0.39, 0.36) | | | .94 | | -0.45  (-0.85, -0.06) | .03 | | -0.36  (-0.77, 0.04) | | | .08 | | | -0.19  (-0.51, 0.12) | .23 | |  |
| CBI | | -0.003  (-0.37, 0.36) | .99 | | 0.08  (-0.29, 0.46) | | .66 | | 0.26  (-0.12, 0.64) | | | .18 | | -0.01  (-0.41, 0.38) | .96 | | 0.02  (-0.38, 0.42) | | | .92 | | | 0.09  (-0.22, 0.41) | .57 | |  |
| **Systolic blood pressure (mm Hg):** F=26.9 *P*<.001; ICC PHCP =0.01; ICC subject|PHCP=0.28 | | | | | | | | | | | | | | | | | | | | | | | | | |  |
| PTI | | -1.7  (-5.2, 1.8) | .33 | | -0.63  (-4.2, 3) | | .73 | | -1.8  (-5.3, 1.8) | | | .32 | | -2.2  (-5.9, 1.4) | .24 | | -3.2  (-6.8, 0.39) | | | .08 | | | -1.9  (-4.5, 0.82) | .17 | |  |
| PFI | | -5.4  (-8.8, -2) | .002 | | 0.06  (-3.4, 3.5) | | .97 | | -0.93  (-4.4, 2.5) | | | .60 | | -3.2  (-6.7, 0.24) | .07 | | -2.0  (-5.5, 1.5) | | | .26 | | | -1.8  (-4.4, 0.73) | .16 | |  |
| CBI | | -1.6  (-5.0, 1.8) | .36 | | 1.1  (-2.4, 4.7) | | .53 | | -1.7  (-5.2, 1.9) | | | .35 | | -2.6  (-6.1, 0.93) | .15 | | -5.2  (-9.0, -1.5) | | | .007 | | | -1.8  (-4.4, 0.76) | .17 | |  |
| **Diastolic blood pressure (mm Hg):** F=22.1 *P*<.001; ICC PHCP =0.02; ICC subject|PHCP=0.23 | | | | | | | | | | | | | | | | | | | | | | | | | |  |
| PTI | | -1.1  (-3.5, 1.3) | .37 | | -1.5  (-3.9, 0.9) | | .22 | | -1.3  (-3.7, 1.1) | | | .29 | | 0.91  (-1.5, 3.3) | .46 | | -1.4  (-3.8, 0.9) | | | .23 | | | -0.71  (-2.6, 1.2) | .46 | |  |
| PFI | | -2.5  (-4.8, -0.25) | .03 | | -1.3  (-3.6, 1.1) | | .29 | | -1.3  (-3.6, 1) | | | .27 | | -0.71  (-3, 1.6) | .55 | | -1.8  (-4.1, 0.52) | | | .13 | | | -1.3  (-3.1, 0.56) | .17 | |  |
| CBI | | -0.03  (-2.4, 2.3) | .98 | | -1.9  (-4.3, 0.43) | | .11 | | -2.4  (-4.8, -0.06) | | | .04 | | -0.99  (-3.3, 1.3) | .41 | | -3.8  (-6.1, -1.4) | | | .002 | | | -1.9  (-3.8, -0.07) | .04 | |  |
| **Waist circumference (cm)**: *F=175.5 P<.001; ICC PHCP =0.02; ICC subject|PHCP=0.44* | | | | | | | | | | | | | | | | | | | | | | | | | |  |
| PTI | | -0.28  (-1.7, 1.2) | .71 | | 0.33  (-1.2, 1.9) | | .68 | | -0.18  (-1.7, 1.4) | | | .82 | | 0.27  (-1.3, 1.8) | .73 | | -0.31  (-1.9, 1.2) | | | .69 | | | 0.03  (-1.2, 1.3) | .96 | |  |
| PFI | | -0.13  (-1.56, 1.3) | .86 | | -2.03  (-3.52, -0.55) | | .007 | | -0.05  (-1.52, 1.41) | | | .94 | | -0.37  (-1.85, 1.1) | .62 | | -1.44  (-2.98, 0.1) | | | .07 | | | -0.77  (-2.0, 0.44) | .21 | |  |
| CBI | | -0.84  (-2.2, 0.56) | .24 | | -0.78  (-2.3, 0.71) | | .31 | | -0.43  (-1.9, 1.1) | | | .57 | | -0.49  (-1.9, 0.99) | .52 | | -0.6  (-2.1, 0.91) | | | .44 | | | -0.58  (-1.8, 0.66) | .36 | |  |
| **Weight (kg)**: *F=484.2 P<.001; ICC PHCP =0.01; ICC subject|PHCP=0.57* | | | | | | | | | | | | | | | | | | | | | | | | | |  |
| PTI | | -0.72  (-1.74, 0.3) | .17 | | -0.53  (-1.57, 0.5) | | .31 | | -0.03  (-1.13, 1.08) | | | .96 | | -1  (-2.14, 0.15) | .09 | | -0.52  (-1.69, 0.65) | | | .38 | | | -0.53  (-1.4, 0.37) | .25 | |  |
| PFI | | -0.33  (-1.32, 0.66) | .0.56 | | -0.23  (-1.22, 0.76) | | .65 | | -0.14  (-1.15, 0.87) | | | .78 | | -1.24  (-2.31, 0.18) | .02 | | -1.07  (-2.15, 0.02) | | | .05 | | | -0.62  (-1.5, 0.22) | .15 | |  |
| CBI | | -0.1  (-1.07, 0.88) | .85 | | 0.16  (-0.83, 1.16) | | .75 | | 0.71  (-0.32, 1.73) | | | .18 | | 0.02  (-1.05, 1.09) | .97 | | 0.08  (-1, 1.16) | | | .88 | | | 0.25  (-0.61, 1.1) | .57 | |  |
| **Waist-to-hip ratio**: *F=42.2 P<.001; ICC PHCP =0.02; ICC subject|PHCP=0.42* | | | | | | | | | | | | | | | | | | | | | | | | | |  |
| PTI | | 0  (-0.01, 0.01) | .98 | | 0  (-0.01, 0.01) | | .98 | | 0.005  (-0.01, 0.02) | | | .39 | | 0.009  (-0.004, 0.02) | .18 | | 0.005  (-0.01, 0.02) | | | .41 | | | 0.005  (-0.01, 0.01) | .37 | |  |
| PFI | | 0.004  (-0.01, 0.02) | .54 | | 0.007  (-0.005, 0.02) | | .27 | | 0.009  (-0.003, 0.02) | | | .16 | | 0.008  (-0.004, 0.02) | .21 | | 0.009  (-0.003, 0.02) | | | .14 | | | 0.008  (-0.002, 0.02) | .13 | |  |
| CBI | | 0.002  (-0.01, 0.01) | .74 | | 0.005  (-0.007, 0.02) | | .40 | | 0.011  (-0.002, 0.02) | | | .098 | | 0.009  (-0.003, 0.02) | .14 | | 0.009  (-0.003, 0.02) | | | .16 | | | 0.008  (-0.002, 0.02) | .12 | |  |
|  | |  |  | | **6M** | | ***P*** | | **12M** | | | ***P*** | |  |  | | **24M** | | | ***P*** | | | **3M to 24M** | ***P*** | |  |
| **Total cholesterol (mg/dL):** F= 43.0 p<.001; ICC PHCP =0.01; ICC subject|PHCP=0.37 | | | | | | | | | | | | | | | | | | | | | | | | | |  |
| PTI | |  |  | | -1.1  (-9.1, 6.8) | | .78 | | 4.8  (-2.8, 12.4) | | | .21 | |  |  | | 1.7  (-6.2, 9.6) | | | .67 | | | 2.4  (-1.6, 6.4) | .24 | |  |
| PFI | |  |  | | -1.3  (-8.9, 6.2) | | .73 | | 2.8  (-4.3, 9.96) | | | .44 | |  |  | | -4.5  (-12.2, 3.2) | | | .25 | | | 0.8  (-3, 4.5) | .69 | |  |
| CBI | |  |  | | -2.4  (-9.9, 5.2) | | .53 | | 3.6  (-3.8, 11) | | | .34 | |  |  | | -2.9  (-10.5, 4.6) | | | .44 | | | 0.9  (-2.9, 4.7) | .65 | |  |
| **LDL^j^ (mg/dL):** F=46.4 p<.001; ICC PHCP =0.01; ICC subject|PHCP=0.35 | | | | | | | | | | | | | | | | | | | | | | | | | |  |
| PTI | |  |  | | 3.5  (-2.9, 10) | | .28 | | 3.9  (-2.5, 10.2) | | | .23 | |  |  | | 2.0  (-4.6, 8.6) | | | .56 | | | 3.3  (0.1, 6.6) | .04 | |  |
| PFI | |  |  | | -4  (-10.1, 2.1) | | .20 | | 2.6  (-3.3, 8.6) | | | .40 | |  |  | | -3.6  (-10, 2.9) | | | .28 | | | -1.1  (-4.2, 1.9) | .47 | |  |
| CBI | |  |  | | -1.7  (-7.9, 4.5) | | .60 | | 3.9  (-2.4, 10.1) | | | .22 | |  |  | | -2.3  (-8.8, 4.2) | | | .49 | | | -0.83  (-3.9, 2.3) | .60 | |  |
| **HDL^k^ (mg/dL):** F=122.6 *P*<.001; ICC PHCP =0.01; ICC subject|PHCP=0.44 | | | | | | | | | | | | | | | | | | | | | | | | | |  |
| PTI | |  |  | | 0.5  (-1.4, 2.3) | | .62 | | 0.1  (-1.7, 2) | | | .88 | |  |  | | 0.6  (-1.3, 2.4) | | | .55 | | | 0.66  (-0.3, 1.6) | .16 | |  |
| PFI | |  |  | | 1.7  (-0.1, 3.4) | | .06 | | 0.1  (-1.6, 1.9) | | | .88 | |  |  | | 1.7  (-0.1, 3.5) | | | .07 | | | 0.95  (0.1, 1.8) | .03 | |  |
| CBI | |  |  | | 1.5  (-0.2, 3.2) | | .09 | | -0.7  (-2.4, 1) | | | .43 | |  |  | | 0.5  (-1.3, 2.4) | | | .57 | | | 1.17  (0.3, 2.0) | .008 | |  |
| **Triglycerides (mg/dL):** F=18.0 *P*<.001; ICC PHCP =0.01; ICC subject|PHCP=0.45 | | | | | | | | | | | | | | | | | | | | | | | | | |  |
| PTI | |  |  | | -14.3  (-29.3, 0.7) | | .06 | | -2.2  (-16.9, 12.6) | | | .77 | |  |  | | -6.8  (-21.7, 8.06) | | | .37 | | | 2.3  (-5.2, 9.8) | .55 | |  |
| PFI | |  |  | | 7.6  (-7.6, 22.8) | | .33 | | -1.1  (-14.8, 12.7) | | | .88 | |  |  | | -10.7  (-24.6, 3.2) | | | .13 | | | 7.64  (0.04, 15.2) | .049 | |  |
| CBI | |  |  | | -1.3  (-16.1, 13.4) | | .86 | | -1.2  (-15.6, 13.1) | | | .87 | |  |  | | 4.3  (-10.9, 19.4) | | | .58 | | | 8.1  (0.7, 15.4) | .03 | |  |
| **Fasting serum glucose** **(mg/dL):** F=21.2 *P*<.001; ICC PHCP =0.01; ICC subject|PHCP=0.28 | | | | | | | | | | | | | | | | | | | | | | | | | |  |
| PTI | |  |  | | -9.6  (-20.2, 0.95) | | .07 | | -2.8  (-13.3, 7.8) | | | .6 | |  |  | | -0.55  (-11.7, 10.6) | | | .92 | | | -4.4  (-9.7, 0.9) | .104 | |  |
| PFI | |  |  | | -15.7  (-25.8, -5.6) | | .002 | | -5.9  (-15.8, 4.0) | | | .24 | |  |  | | -0.55  (-11.1, 10.0) | | | .92 | | | -9.8  (-14.9, -4.7) | <.001 | |  |
| CBI | |  |  | | -13.0  (-23, -2.9) | | .01 | | -7.2  (-17.2, 2.9) | | | .17 | |  |  | | -1.4  (-12.2, 9.4) | | | .80 | | | -7.2  (-12.2, -2.16) | .005 | |  |
|  | |  |  | |  | |  | | **12M** | | | ***P*** | |  |  | | **24M** | | | ***P*** | | | **12M to 24M** | ***P*** | |  |
| **Serum** **Creatinine (mg/dL):** F=164.6 *P*<.001; ICC PHCP =0.17; ICC subject|PHCP=0.48 | | | | | | | | | | | | | | | | | | | | | | | | | |  |
| PTI | |  |  | |  | |  | | -0.02  (-0.08, 0.05) | | | .66 | |  |  | | -0.04  (-0.1, 0.03) | | | .25 | | | -0.03  (-0.09, 0.04) | .42 | |  |
| PFI | |  |  | |  | |  | | -0.03  (-0.1, 0.03) | | | .33 | |  |  | | -0.04  (-0.1, 0.02) | | | .21 | | | -0.04  (-0.1, 0.03) | .26 | |  |
| CBI | |  |  | |  | |  | | 0.02  (-0.04, 0.09) | | | .53 | |  |  | | 0.002  (-0.06, 0.07) | | | .96 | | | 0.01  (-0.05, 0.08) | .73 | |  |
| **Glomerular filtration rate (mL/min):** F=128.6 *P*<.001; ICC PHCP =0.21; ICC subject|PHCP=0.54 | | | | | | | | | | | | | | | | | | | | | | | | | |  |
| PTI | |  |  | |  | |  | | 1.2  (-7.2, 9.5) | | | .78 | |  |  | | 4.7  (-3.7, 13.1) | | | .27 | | | 3.0  (-5.2, 11.1) | .48 | |  |
| PFI | |  |  | |  | |  | | 2.9  (-5.4, 11.2) | | | .49 | |  |  | | 5.7  (-2.7, 14.1) | | | .18 | | | 4.3  (-3.9, 12.5) | .30 | |  |
| CBI | |  |  | |  | |  | | -4.3  (-12.6, 4.0) | | | .31 | |  |  | | 0.19  (-8.2, 8.6) | | | .96 | | | -2.0  (-10.3, 6.2) | .63 | |  |
| ^a^UC: usual care or control group.  ^b^AUC: area under the curve.  ^c^M: months.  ^d^HbA_1c_: glycated hemoglobin.  ^e^ICC: Intraclass correlation coefficient.  ^f^PHCP: Primary Care Health Practices.  ^g^PTI is an intervention only for patients and family members.  ^h^PFI is an intervention only for health care professionals at primary care.  ^i^CBI is a combined intervention for patients and professionals.  ^j^LDL: low-density lipoprotein.  ^k^HDL: high-density lipoprotein. | | | | | | | | | | | | | | | | | | | | | | | | | |  |
