# Supplementary material for: Effectiveness of Internet-Based Multicomponent Interventions for Patients and Health Care Professionals to Improve Clinical Outcomes in Type 2 Diabetes Evaluated Through the INDICA Study: Multiarm Cluster Randomized Controlled Trial
Source: JMIR Mhealth Uhealth. 2020 Nov 2;8(11):e18922. doi: 10.2196/18922 (PMC7669446; doi:10.2196/18922)
Supplement: Multimedia Appendix 8 [file mhealth_v8i11e18922_app8.docx]

Multimedia Appendix 8. Adjusted means for each group and intragroup differences compared with the baseline measurement for patients with a baseline HbA_1c_ >7. HbA_1c_: glycated hemoglobin.

|  | | **Adjusted means in each group (95%CI)** | | | | | | | | | **Difference in intragroup adjusted means compared to baseline (95%CI)** | | | | | | | |
| --- | --- | --- | --- | --- | --- | --- | --- | --- | --- | --- | --- | --- | --- | --- | --- | --- | --- | --- |
|  | | **B^d^** | **3M^e^** | **6M** | **12M** | | **18M** | | **24M** | | **3M-B** | **6M-B** | **12M-B** | **18M-B** | | **24M-B** | | |
| **HbA_1c_^f^ (%)** | | | | | | | | | | | | | | | | | | |
| PTI^g^ | | 8.4  (8.3, 8.5) | 7.7  (7.5, 8.0) | 7.8  (7.6, 8.0) | 7.9  (7.7, 8.1) | | 8.1  (7.9, 8.4) | | 8.0  (7.7, 8.2) | | -0.69  (-0.9, -0.5)^a^ | -0.61  (-0.8, -0.4)^a^ | -0.56  (-0.8, -0.3)^a^ | -0.30  (-0.5, -0.08)^b^ | | | | -0.49  (-0.7, -0.3)^a^ |
| PFI^h^ | | 8.3  (8.3, 8.4) | 8.0  (7.8, 8.2) | 8.0  (7.7, 8.2) | 8.1  (7.9, 8.3) | | 8.0  (7.8, 8.2) | | 8.0  (7.8, 8.2) | | -0.36  (-0.6, -0.2)^b^ | -0.38  (-0.6, -0.2)^a^ | -0.21  (-0.4,-0.002)^b^ | -0.38  (-0.6, -0.2)^a^ | | | | -0.35  (-0.6, -0.4)^b^ |
| CBI^i^ | | 8.4  (8.3, 8.6) | 8.0  (7.8, 8.2) | 8.0  (7.8, 8.2) | 7.9  (7.7, 8.1) | | 8.0  (7.7, 8.1) | | 8.0  (7.8, 8.2) | | -0.38  (-0.6, -0.2)^a^ | -0.38  (-0.6, -0.2)^a^ | -0.45  (-0.7, -0.3)^a^ | -0.43  (-0.7, -0.2)^a^ | | | | -0.35  (-0.6, -0.1)^b^ |
| UC^j^ | | 8.5  (8.4, 8.6) | 8.3  (8.1, 8.5) | 8.3  (8.1, 8.5) | 8.2  (8.0, 8.4) | | 8.2  (8.0, 8.4) | | 8.1  (7.8, 8.3) | | -0.20  (-0.4, 0.003)^c^ | -0.16  (-0.4, 0.05) | -0.30  (-0.5, -0.08)^b^ | -0.27  (-0.5, -0.05)^b^ | | | | -0.41  (-0.6, -0.2)^a^ |
| **BMI (Kg/m^2^)** | | |  |  |  | |  | |  | |  |  |  |  | |  | | |
| PTI | 32.2  (31.9, 32.5) | | 32  (31.7, 32.3) | 32  (31.7, 32.3) | | 31.9  (31.6, 32.3) | | 31.9  (31.5, 32.2) | | 31.8  (31.5, 32.2) | -0.21  (-0.49, 0.07) | -0.16  (-0.45, 0.12) | -0.24  (-0.56, 0.07) | | -0.33  (-0.65, 0)^b^ | | -0.38  (-0.73, -0.04)^b^ | |
| PFI | 32.9  (32.6, 33.2) | | 32.2  (31.9, 32.4) | 32.2  (31.9, 32.5) | | 31.9  (31.7, 32.2) | | 31.8  (31.5, 32.1) | | 31.7  (31.4, 31.9) | -0.76  (-1.02, -0.5)^a^ | -0.74  (-1, -0.48)^a^ | -0.99  (-1.25, -0.72)^a^ | | -1.12  (-1.4, -0.83)^a^ | | -1.28  (-1.57, -0.99)^a^ | |
| CBI | 32  (31.7, 32.3) | | 32.2  (32, 32.5) | 32.3  (32, 32.6) | | 32.2  (31.9, 32.5) | | 32.3  (32, 32.5) | | 32  (31.7, 32.3) | 0.23  (-0.03, 0.48)^c^ | 0.3  (0.04, 0.57)^b^ | 0.23  (-0.05, 0.5) | | 0.27  (-0.02, 0.55)c | | 0.04  (-0.25, 0.34)^b^ | |
| UC | 32.1  (31.7, 32.4) | | 32.2  (32, 32.5) | 32.2  (32, 32.5) | | 32  (31.7, 32.2) | | 32.3  (32, 32.6) | | 32  (31.7, 32.3) | 0.14  (-0.12, 0.4) | 0.13  (-0.13, 0.39) | -0.12  (-0.39, 0.15) | | 0.19  (-0.09, 0.47) | | -0.06  (-0.34, 0.21) | |
| **Systolic blood pressure (mm Hg)** | | | | | | | | | |  |  |  |  | |  | |  | |
| PTI | 135.1  (134.1, 136.1) | | 130.8  (128.2, 133.3) | 132.8  (130.2, 135.4) | | 130.6  (128, 133.2) | | 132  (129.3, 134.7) | | 128.7  (126, 131.3) | -4.3  (-6.8, -1.8)^b^ | -2.3  (-4.9, 0.28)^c^ | -4.5  (-7.1, -1.9)^b^ | | -3.1  (-5.8, -0.42)^b^ | | -6.4  (-9, -3.8)^a^ | |
| PFI | 136.5  (135.6, 137.5) | | 127.1  (124.7, 129.5) | 133.5  (131.1, 135.8) | | 131.4  (129.1, 133.8) | | 131  (128.6, 133.4) | | 129.9  (127.4, 132.4) | -9.4  (-11.8, -7.0)^a^ | -3.1  (-5.4, -0.68)^b^ | -5.1  (-7.5, -2.7)^a^ | | -5.5  (-7.9, -3.2)^a^ | | -6.6  (-9.1, -4.2)^a^ | |
| CBI | 135.5  (134.3, 136.6) | | 130.9  (128.5, 133.3) | 134.6  (132.1, 137) | | 130.7  (128.2, 133.2) | | 131.6  (129.2, 134.1) | | 126.7  (123.7, 129.7) | -4.6  (-7, -2.2)^a^ | -0.9  (-3.4, 1.47) | -4.8  (-7.3, -2.3)^a^ | | -3.9  (-6.3, -1.4)^b^ | | -8.8  (-11.8, -5.8)^a^ | |
| UC | 134.4  (133.4, 135.4) | | 132.5  (130.1, 134.9) | 133.4  (130.9, 135.9) | | 132.4  (129.9, 134.8) | | 134.2  (131.7, 136.7) | | 131.9  (129.5, 134.3) | -1.9  (-4.3, 0.49) | -1  (-3.5, 1.49) | -2  (-4.5, 0.41) | | -0.2  (-2.7, 2.26) | | -2.5  (-4.9, -0.13)^b^ | |
| **Diastolic blood pressure (mm Hg)** | | | | | |  | |  | |  |  |  |  | |  | |  | |
| PTI | 85.2  (84.7, 85.8) | | 82  (80.3, 83.7) | 83.5  (81.9, 85.2) | | 81.7  (80.1, 83.4) | | 83.8  (82, 85.5) | | 81.3  (79.6, 83) | -3.3  (-5, -1.6)^a^ | -1.7  (-3.4, 0)^c^ | -2.4  (-4.1, -0.63)^b^ | | -1.5  (-3.2, 0.24)^c^ | | -4  (-5.7, -2.3)^a^ | |
| PFI | 85.4  (84.8, 85.9) | | 80.5  (78.9, 82.1) | 83.8  (82.1, 85.4) | | 82.9  (81.2, 84.5) | | 82.1  (80.5, 83.8) | | 80.9  (79.2, 82.6) | -4.9  (-6.5, -3.2)^a^ | -1.6  (-3.2, 0.04)^c^ | -2.5  (-4.1, -0.87)^b^ | | -3.2  (-4.9, -1.6)^a^ | | -4.4  (-6.1, -2.7)^a^ | |
| CBI | 86.2  (85.5, 86.8) | | 83  (81.3, 84.7) | 83.1  (81.5, 84.7) | | 82.9  (81.2, 84.6) | | 81.9  (80.2, 83.5) | | 78.9  (77.2, 80.6) | -3.2  (-4.9, -1.5)^a^ | -3.1  (-4.7, -1.4)^a^ | -4.4  (-6.1, -2.79)^a^ | | -4.3  (-6, -2.67)^a^ | | -7.3  (-9, -5.57)^a^ | |
| UC | 84  (83.5, 84.6) | | 83  (81.4, 84.7) | 85  (83.4, 86.7) | | 84.2  (82.5, 85.8) | | 82.8  (81.2, 84.5) | | 82.7  (81.1, 84.3) | -1  (-2.6, 0.63) | 1  (-0.7, 2.65) | 0.1  (-1.5, 1.76) | | -1.2  (-2.8, 0.46) | | -1.3  (-2.9, 0.28) | |
| **Waist circumference (cm)** | | | | | |  | |  | |  |  |  |  | |  | |  | |
| PTI | 106.8  (106.1, 107.5) | | 107  (105.9, 108) | 107.5  (106.4, 108.7) | | 106.7  (105.6, 107.8) | | 106.9  (105.8, 108) | | 106.4  (105.2, 107.6) | 0.18  (-0.87, 1.23) | 0.76  (-0.38, 1.89) | -0.07  (-1.2, 1.06) | | 0.14  (-0.99, 1.27) | | -0.38  (-1.55, 0.8) | |
| PFI | 109  (108.4, 109.7) | | 107.1  (106.1, 108.1) | 105.2  (104.1, 106.2) | | 106.8  (105.8, 107.9) | | 106.3  (105.2, 107.3) | | 105.3  (104.2, 106.4) | -1.91  (-2.92, -0.9)^a^ | -3.85  (-4.91, -2.8)^a^ | -2.19  (-3.23, -1.14)^a^ | | -2.74  (-3.8, -1.68)^a^ | | -3.74  (-4.84, -2.64)^a^ | |
| CBI | 106.2  (105.6, 106.9) | | 106.4  (105.4, 107.4) | 106.4  (105.4, 107.5) | | 106.4  (105.4, 107.5) | | 106.2  (105.1, 107.2) | | 106.1  (105, 107.2) | 0.18  (-0.81, 1.17) | 0.2  (-0.85, 1.25) | 0.23  (-0.83, 1.3) | | -0.05  (-1.1, 1) | | -0.1  (-1.19, 0.99) | |
| UC | 106.4  (105.7, 107.1) | | 107.2  (106.2, 108.2) | 107.2  (106.2, 108.2) | | 106.9  (105.8, 107.9) | | 106.6  (105.6, 107.7) | | 106.7  (105.7, 107.8) | 0.84  (-0.17, 1.85) | 0.81  (-0.24, 1.85) | 0.49  (-0.55, 1.53) | | 0.26  (-0.78, 1.3) | | 0.32  (-0.73, 1.37) | |
| **Weight (kg)** | | | | | |  | |  | |  |  |  |  | |  | |  | |
| PTI | 88.5  (86.4, 90.5) | | 87.1  (86.4, 87.9) | 87.3  (86.5, 88) | | 87  (86.2, 87.9) | | 86.8  (85.9, 87.7) | | 86.7  (85.7, 87.6) | -0.83  (-1.58, -0.08)^b^ | -0.69  (-1.46, 0.08)^c^ | -0.91  (-1.78, -0.05)^b^ | | -1.12  (-2.01, -0.24)^b^ | | -1.28  (-2.22, -0.34)^b^ | |
| PFI | 89.4  (87.4, 91.3) | | 87.5  (86.8, 88.2) | 87.6  (86.8, 88.3) | | 86.9  (86.2, 87.6) | | 86.6  (85.8, 87.3) | | 86.1  (85.3, 86.9) | -0.44  (-1.13, 0.25) | -0.38  (-1.09, 0.33) | -1.03  (-1.75, -0.31)^b^ | | -1.37  (-2.14, -0.61)^a^ | | -1.83  (-2.61, -1.04)^a^ | |
| CBI | 87.3  (85.4, 89.2) | | 87.7  (87.1, 88.4) | 88  (87.2, 88.7) | | 87.8  (87, 88.5) | | 87.8  (87, 88.6) | | 87.3  (86.5, 88.1) | -0.2  (-0.89, 0.48) | 0.01  (-0.7, 0.72) | -0.18  (-0.92, 0.56) | | -0.11  (-0.89, 0.67) | | -0.68  (-1.47, 0.11)^c^ | |
| UC | 86.6  (84.5, 88.8) | | 87.8  (87.1, 88.5) | 87.8  (87.1, 88.5) | | 87.1  (86.3, 87.8) | | 87.8  (87, 88.6) | | 87.2  (86.4, 87.9) | -0.11  (-0.81, 0.59) | -0.15  (-0.85, 0.55) | -0.89  (-1.61, -0.17)^b^ | | -0.13  (-0.89, 0.63) | | -0.76  (-1.51, -0.01)^b^ | |
| **Waist-to-hip ratio** | | | | | |  | |  | |  |  |  |  | |  | |  | |
| PTI | 1  (0.99, 1) | | 0.99  (0.98, 1) | 0.99  (0.98, 1) | | 0.99  (0.98, 1) | | 1  (0.99, 1.01) | | 1  (0.99, 1) | -0.009  (-0.02, 0) | -0.008  (-0.02, 0) | -0.002  (-0.01, 0.01) | | 0.003  (-0.006, 0.01) | | 0  (-0.009, 0.01) | |
| PFI | 0.99  (0.98, 0.99) | | 0.99  (0.98, 1) | 0.99  (0.99, 1) | | 1  (0.99, 1.01) | | 1  (0.99, 1.01) | | 1  (0.99, 1.01) | 0.004  (-0.004, 0.01) | 0.008  (0, 0.02) | 0.011  (0.002, 0.02) | | 0.011  (0.003, 0.02) | | 0.013  (0.004, 0.02) | |
| CBI | 0.99  (0.99, 0.99) | | 0.99  (0.98, 1) | 0.99  (0.98, 1) | | 1  (0.99, 1.01) | | 1  (0.99, 1.01) | | 1  (0.99, 1.01) | 0  (-0.009, 0.01) | 0.004  (-0.005, 0.01) | 0.01  (0.001, 0.02) | | 0.01  (0.001, 0.02) | | 0.01  (0.001, 0.02) | |
| UC | 1  (1, 1.01) | | 0.99  (0.98, 0.99) | 0.99  (0.98, 1) | | 0.99  (0.98, 1) | | 0.99  (0.98, 1) | | 0.99  (0.98, 1) | -0.02  (-0.02, -0.01) | -0.01  (-0.02, -0.01) | -0.01  (-0.02, -0.01) | | -0.01  (-0.02, 0) | | -0.01  (-0.02, 0) | |
|  | | **B** |  | **6M** | **12M** | |  | | **24M** | |  | **6M-B** | **12M-B** |  | | **24M-B** | | |
| **Total cholesterol (mg/dL)** | | | | | |  | |  | |  |  |  |  | |  | |  | |
| PTI | 192  (189.7, 194.2) | |  | 184.9  (179.2, 190.7) | | 187.2  (181.7, 192.7) | |  | | 183.6  (177.9, 189.3) |  | -7.1  (-12.8, -1.3)^b^ | -4.8  (-10.3, 0.71)^c^ | |  | | -8.4  (-14.1, -2.65)^b^ | |
| PFI | 188.9  (186.6, 191.3) | |  | 184.7  (179.5, 190) | | 185.2  (180.2, 190.2) | |  | | 177.4  (172.1, 182.7) |  | -4.2  (-9.5, 1.08) | -3.7  (-8.7, 1.24) | |  | | -11.6  (-16.9, -6.25)^a^ | |
| CBI | 190.2  (187.9, 192.6) | |  | 183.7  (178.4, 188.9) | | 186  (180.7, 191.3) | |  | | 178.9  (173.5, 184.4) |  | -6.5  (-11.8, -1.31)^b^ | -4.2  (-9.5, 1.06) | |  | | -11.3  (-16.7, -5.85)^a^ | |
| UC | 186.1  (183.8, 188.4) | |  | 186.1  (180.6, 191.5) | | 182.4  (177.2, 187.5) | |  | | 181.9  (176.5, 187.3) |  | -0.01  (-5.5, 5.45) | -3.7  (-8.8, 1.42) | |  | | -4.2  (-9.6, 1.17) | |
| **LDL^k^ (mg/dL)** | | | | | |  | |  | |  |  |  |  | |  | |  | |
| PTI | 110.5  (108.5, 112.6) | |  | 46.6  (45.2, 47.9) | | 105.9  (101.3, 110.4) | |  | | 104.7  (99.9, 109.4) |  | -0.17  (-4.8, 4.48) | -4.7  (-9.2, -0.1)^b^ | |  | | -5.9  (-10.6, -1.12)^b^ | |
| PFI | 109.2  (107.1, 111.3) | |  | 47.8  (46.6, 49) | | 104.6  (100.5, 108.8) | |  | | 99.1  (94.7, 103.6) |  | -6.3  (-10.6, -2)^b^ | -4.5  (-8.7, -0.35)^b^ | |  | | -10.0  (-14.4, -5.61)^a^ | |
| CBI | 107.5  (105.4, 109.6) | |  | 47.6  (46.4, 48.9) | | 105.9  (101.4, 110.3) | |  | | 100.4  (95.8, 105.1) |  | -2.3  (-6.7, 2.06) | -1.6  (-6, 2.88) | |  | | -7.0  (-11.7, -2.36)^b^ | |
| UC | 107.5  (105.4, 109.5) | |  | 46.1  (44.9, 47.4) | | 102  (97.7, 106.4) | |  | | 102.7  (98.2, 107.2) |  | -0.64  (-5, 3.76) | -5.4  (-9.8, -1.1)^b^ | |  | | -4.8  (-9.3, -0.24)^b^ | |
| **HDL^l^ (mg/dL)** | | | | | |  | |  | |  |  |  |  | |  | |  | |
| PTI | 47.3  (46.7, 48) | |  | 46.6  (45.2, 47.9) | | 47  (45.7, 48.3) | |  | | 46.3  (45, 47.7) |  | -0.73  (-2.1, 0.61) | -0.34  (-1.7, 0.99) | |  | | -0.99  (-2.4, 0.37) | |
| PFI | 46.7  (46, 47.4) | |  | 47.8  (46.6, 49) | | 47  (45.8, 48.2) | |  | | 47.5  (46.2, 48.7) |  | 1.1  (-0.1, 2.4)^c^ | 0.28  (-0.9, 1.5) | |  | | 0.77  (-0.5, 2.05) | |
| CBI | 47.3  (46.6, 47.9) | |  | 47.6  (46.4, 48.9) | | 46.1  (44.9, 47.4) | |  | | 46.3  (45, 47.6) |  | 0.36  (-0.9, 1.6) | -1.2  (-2.4, 0.11)^c^ | |  | | -0.98  (-2.3, 0.31) | |
| UC | 46.5  (45.8, 47.1) | |  | 46.1  (44.9, 47.4) | | 46.8  (45.6, 48.1) | |  | | 45.8  (44.5, 47) |  | -0.32  (-1.6, 0.92) | 0.38  (-0.9, 1.6) | |  | | -0.69  (-2, 0.59) | |
| **Triglycerides (mg/dL)** | | | | | |  | |  | |  |  |  |  | |  | |  | |
| PTI | 185.7  (178.2, 193.1) | |  | 161.4  (143.1, 179.7) | | 183.4  (165.6, 201.2) | |  | | 166  (148.4, 183.5) |  | -24.3  (-42.6, -5.97)^b^ | -2.3  (-20.1, 15.47) | |  | | -19.7  (-37.3, -2.19)^b^ | |
| PFI | 174.5  (168.6, 180.5) | |  | 185.2  (166.8, 203.5) | | 178.6  (162.4, 194.8) | |  | | 165.8  (149.2, 182.3) |  | 10.7  (-7.7, 29.02) | 4.1  (-12.1, 20.3) | |  | | -8.8  (-25.4, 7.83) | |
| CBI | 184.3  (177.8, 190.7) | |  | 176.7  (160.5, 192.9) | | 182.2  (166, 198.3) | |  | | 179.1  (161.9, 196.4) |  | -7.5  (-23.7, 8.67) | -2.1  (-18.3, 14.05) | |  | | -5.1  (-22.3, 12.09) | |
| UC | 166.8  (159.9, 173.7) | |  | 177.5  (159.6, 195.4) | | 173.6  (156.9, 190.3) | |  | | 171.8  (154.9, 188.7) |  | 10.7  (-7.2, 28.55) | 6.8  (-10, 23.49) | |  | | 5.0  (-11.9, 21.88) | |
| **Fasting serum glucose (mg/dL)** | | | | | |  | |  | |  |  |  |  | |  | |  | |
| PTI | 182.1  (179, 185.2) | |  | 173.8  (166.2, 181.4) | | 173  (165.2, 180.7) | |  | | 170.1  (162.1, 178.2) |  | -8.3  (-15.9, -0.69)^b^ | -9.1  (-16.8, -1.34)^b^ | |  | | -11.9  (-20, -3.91)^b^ | |
| PFI | 177.3  (174.4, 180.2) | |  | 167.7  (160.6, 174.8) | | 169.9  (163, 176.8) | |  | | 170.1  (162.7, 177.5) |  | -9.6  (-16.7, -2.5)^b^ | -7.5  (-14.3, -0.54)^b^ | |  | | -7.2  (-14.6, 0.22)^c^ | |
| CBI | 179.8  (176.7, 182.9) | |  | 170.5  (163.4, 177.5) | | 168.6  (161.6, 175.6) | |  | | 169.3  (161.7, 176.8) |  | -9.3  (-16.4, -2.26)^b^ | -11.2  (-18.2, -4.17)^b^ | |  | | -10.5  (-18.1, -2.94)^b^ | |
| UC | 181.2  (178.1, 184.3) | |  | 183.4  (176.1, 190.7) | | 175.8  (168.6, 182.9) | |  | | 170.7  (163.2, 178.2) |  | 2.2  (-5.1, 9.53) | -5.5  (-12.6, 1.71) | |  | | -10.5  (-18, -3.04)^b^ | |
|  | **B** | |  |  | | **12M** | |  | | **24M** |  |  | **12M-B** | |  | | **24M-B** | |
| **Serum Creatinine (mg/dL)** | | | | | |  | |  | |  |  |  |  | |  | |  | |
| PTI | 0.81  (0.8, 0.82) | |  |  | | 0.78  (0.73, 0.82) | |  | | 0.78  (0.73, 0.82) |  |  | -0.03  (-0.1, 0.01) | |  | | -0.04  (-0.1, 0.01) | |
| PFI | 0.8  (0.79, 0.81) | |  |  | | 0.76  (0.71, 0.81) | |  | | 0.77  (0.73, 0.82) |  |  | -0.04  (-0.1, 0.01)^c^ | |  | | -0.03  (-0.1, 0.02) | |
| CBI | 0.77  (0.76, 0.78) | |  |  | | 0.81  (0.77, 0.86) | |  | | 0.82  (0.77, 0.86) |  |  | 0.04  (0, 0.09)^c^ | |  | | 0.04  (0, 0.09)^c^ | |
| UC | 0.76  (0.75, 0.77) | |  |  | | 0.79  (0.75, 0.84) | |  | | 0.81  (0.77, 0.86) |  |  | 0.03  (0, 0.08) | |  | | 0.05  (0, 0.1)^b^ | |
| **Glomerular filtration rate (mL/min)** | | | | | |  | |  | |  |  |  |  | |  | |  | |
| PTI | 92.1  (90.8, 93.4) | |  |  | | 95.4  (89.5, 101.3) | |  | | 94.5  (88.6, 100.4) |  |  | 3.3  (-2.6, 9.2) | |  | | 2.4  (-3.5, 8.3) | |
| PFI | 90.3  (89.1, 91.5) | |  |  | | 97.1  (91.3, 103) | |  | | 95.5  (89.6, 101.4) |  |  | 6.8  (1, 12.7)^b^ | |  | | 5.2  (-0.7, 11.1)^c^ | |
| CBI | 95.6  (94.5, 96.8) | |  |  | | 89.9  (84, 95.8) | |  | | 90.0  (84.1, 96) |  |  | -5.8  (-11.7, 0.16)^c^ | |  | | -5.6  (-11.6, 0.31)^c^ | |
| UC | 97.2  (95.8, 98.5) | |  |  | | 94.2  (88.3, 100.1) | |  | | 89.8  (83.9, 95.8) |  |  | -3  (-8.9, 3) | |  | | -7.3  (-13.3, -1.4)^b^ | |
| ^a^: *P*<.001.  ^b^: *P*<.05.  ^c^: *P*<.1.  ^d^B: baseline.  ^e^M: months.  ^f^HbA_1c_: glycated hemoglobin.  ^g^PTI is an intervention only for patients and family members.  ^h^PFI is an intervention only for health care professionals at primary care.  ^i^CBI is a combined intervention for patients and professionals.  ^j^UC: usual care or control group.  ^k^LDL: low-density lipoprotein.  ^l^HDL: high-density lipoprotein. | | | | | | | | | | | | | | | | | | |
|  | | | |  |  | |  | |  | |  |  |  |  | |  | | |
